# CASK modulates the assembly and function of the Mint1/Munc18-1 complex to regulate insulin secretion

**DOI:** 10.1038/s41421-020-00216-3

**Published:** 2020-12-15

**Authors:** Zhe Zhang, Wei Li, Guang Yang, Xuefeng Lu, Xin Qi, Shuting Wang, Can Cao, Peng Zhang, Jinqi Ren, Jiaxu Zhao, Junyi Zhang, Sheng Hong, Yan Tan, James Burchfield, Yang Yu, Tao Xu, Xuebiao Yao, David James, Wei Feng, Zhengjun Chen

**Affiliations:** 1 State Key Laboratory of Cell Biology, CAS Center for Excellence in Molecular Cell Science, Institute of Biochemistry and Cell Biology, Chinese Academy of Sciences, Shanghai, 200031 China; 2 National Laboratory of Biomacromolecules, CAS Center for Excellence in Biomacromolecules, Institute of Biophysics, Chinese Academy of Sciences, Beijing, 100101 China; 3 Charles Perkins Centre, School of Life and Environmental Sciences, The University of Sydney, Sydney, NSW 2006 Australia; 4 College of Life Sciences, University of Chinese Academy of Sciences, Beijing, 100049 China; 5 School of Life Sciences and Technology, ShanghaiTech University, Shanghai, 201210 China; 6 National Center for Protein Science Shanghai, Institute of Biochemistry and Cell Biology, Shanghai Institutes for Biological Sciences, Chinese Academy of Sciences, Shanghai, 200031 China; 7 Anhui Key Laboratory for Cellular Dynamics and Chemical Biology, Hefei National Laboratory for Physical Sciences at Nanoscale, University of Science and Technology of China, Hefei, Anhui 230026 China; 8 Sydney Medical School, The University of Sydney, Sydney, NSW 2006 Australia

**Keywords:** Membrane fusion, Cell signalling

## Abstract

Calcium/calmodulin-dependent protein serine kinase (CASK) is a key player in vesicle transport and release in neurons. However, its precise role, particularly in nonneuronal systems, is incompletely understood. We report that CASK functions as an important regulator of insulin secretion. CASK depletion in mouse islets/β cells substantially reduces insulin secretion and vesicle docking/fusion. CASK forms a ternary complex with Mint1 and Munc18-1, and this event is regulated by glucose stimulation in β cells. The crystal structure of the CASK/Mint1 complex demonstrates that Mint1 exhibits a unique “whip”-like structure that wraps tightly around the CASK-CaMK domain, which contains dual hydrophobic interaction sites. When triggered by CASK binding, Mint1 modulates the assembly of the complex. Further investigation revealed that CASK-Mint1 binding is critical for ternary complex formation, thereby controlling Munc18-1 membrane localization and insulin secretion. Our work illustrates the distinctive molecular basis underlying CASK/Mint1/Munc18-1 complex formation and reveals the importance of the CASK-Mint1-Munc18 signaling axis in insulin secretion.

## Introduction

Insulin is secreted by pancreatic β cells mainly in response to elevated blood glucose levels. The process of insulin secretion is tightly regulated in mammals. Pancreatic β cells secrete insulin via Ca^2+^-dependent exocytosis of secretory granules. Soluble N-ethylmaleimide sensitive factor attachment protein receptor (SNARE) complexes are essential for β cell exocytosis, which shares similar basic fusion machinery for exocytosis with complexes controlling neurotransmitter release and is dependent on close association with L-type Ca^2+^ channels^1,2^. Serial steps in SNARE complex assembly and zipping are required for vesicle docking and fusion^3,4^. However, in a physiological context, SNARE complex assembly alone does not mediate fusion. Munc18-1 and Munc13-1 orchestrate SNARE complex formation and play critical roles in vesicle fusion and release^5,6^. Conformational changes in the Sec1/Munc18 (SM) proteins have been proposed to be important for facilitating SNARE complex assembly^7,8^. However, the mechanism of this key process awaits further clarification.

Calcium/calmodulin-dependent protein serine kinase (CASK), an evolutionarily conserved multidomain scaffolding protein belonging to the membrane-associated guanylate kinase (MAGUK) family, is an important regulator of vesicle transport and neurotransmitter release in neurons^9^. In addition to the signature MAGUK motifs, the guanylate kinase-like (GUK) motif, and the PDZ and SH3 domains, CASK also contains two L27 domains and a CaMK-like domain at the N-terminus. Each domain mediates specific protein–protein interactions^9^. Several binding partners of CASK are directly involved in vesicle exocytosis. For instance, CASK functions as a scaffold protein to form a complex with Munc18-1-interacting protein 1 (Mint1)/Veli, acting as a nucleation site for the assembly of proteins involved in synaptic vesicle exocytosis^10^ or with MALS/Liprin-α to regulate neurotransmitter release^11^. CASK also interacts with and regulates the synaptic targeting of N-type calcium channels. Consistent with this function, knockdown of CASK inhibits synaptic transmission in *Lymnaea stagnalis*^12^, and in CASK-deficient neurons, the rate of spontaneous synaptic vesicle release events is changed. However, the mechanism by which CASK regulates its binding proteins to directly affect vesicle release—in particular, docking and fusion—is incompletely characterized. CASK is thus an important regulator in synaptic vesicle release via an unclear mechanism. In addition to being highly expressed in neurons, CASK is also abundantly expressed in peripheral tissues. Determining whether CASK plays a role in other secretory cells, particularly pancreatic β cells, would thus be interesting.

Here, we explored the functional significance of CASK in insulin secretion in β cells. We found that β cell-specific knockout (KO) of CASK in mouse islets strongly reduced glucose-induced insulin secretion. The results of electron microscopy and total internal reflection fluorescence microscopy (TIRFM) suggested that CASK is required for vesicle docking and both the mode and frequency of insulin granule fusion. We further demonstrated the importance of glucose-induced CASK/Mint1/Munc18-1 ternary complex formation in insulin release. The crystal structure of the CASK/Mint1 complex revealed a unique interaction interface between the CASK interaction domain in Mint1 (Mint1-CID) and the CaMK domain in CASK (CASK-CaMK). Disruption of the CASK-Mint1 interaction notably impaired ternary complex formation, Munc18-1 membrane localization and insulin secretion. Our findings suggest that CASK plays a direct role in insulin secretion.

## Results

### CASK is required for insulin secretion by regulating vesicle docking and fusion

**Fig. 1:**
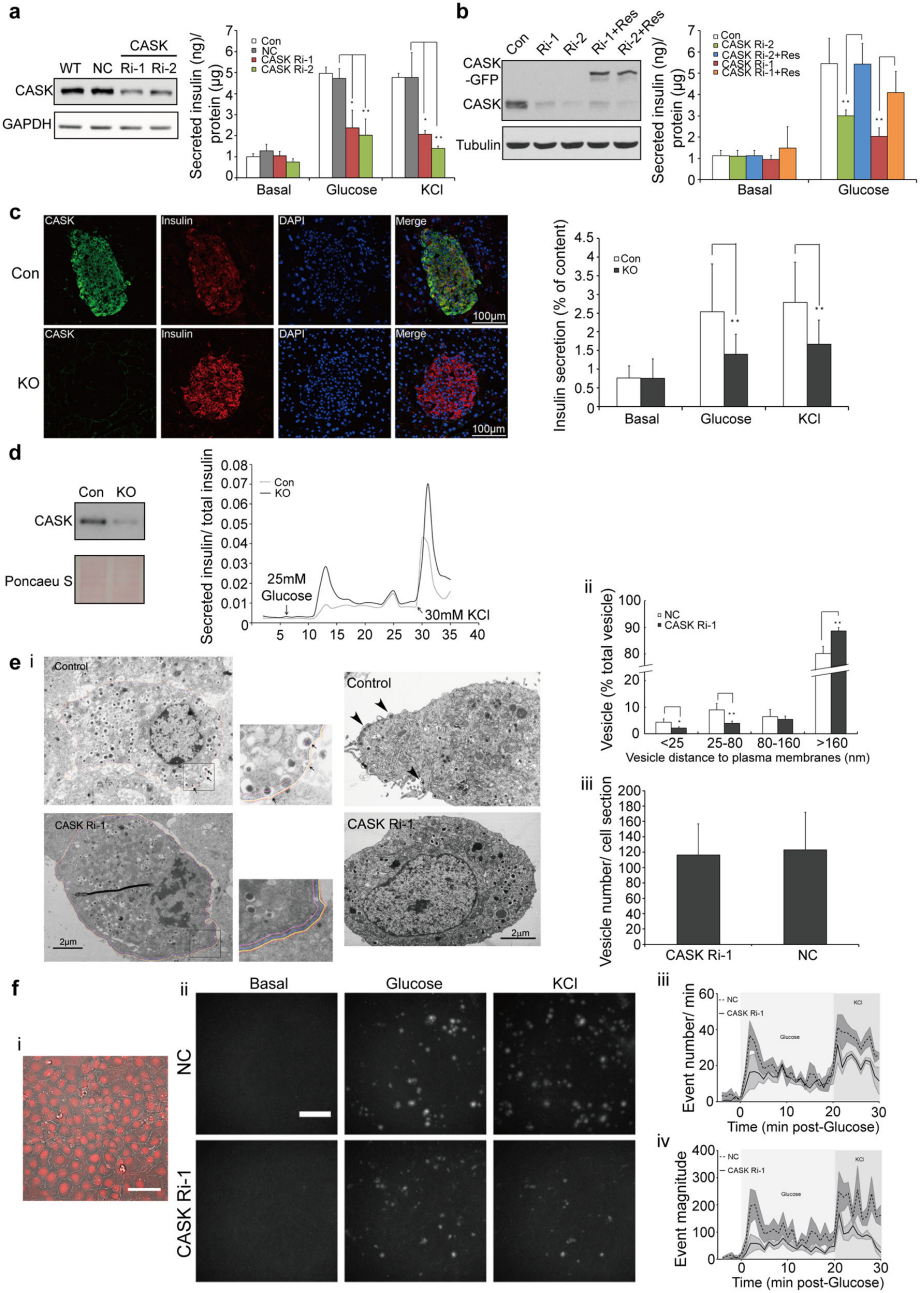
CASK regulates insulin vesicle docking and fusion. **a**, **b** Insulin secretion in INS-1E cells treated with glucose or KCl. CASK was knocked down using two distinct siRNA fragments (Ri-1 or Ri-2). Non-silencing control siRNA (NC) was used as control. WT, wild type cells; Con, empty vector and NC siRNA transfected cells; Res, siRNA-resistant CASK. Western blotting showing the efficiency of CASK knockdown and the expression levels of siRNA-resistant CASK. **c** Insulin secretion in KO islets. Left panel, fixed islet sections were stained with anti-CASK (green), anti-insulin (red, β cells) and DAPI (DNA, blue). Right panel, insulin secretion in the isolated primary islets from the control mice with RIP-Cre and the CASK β-cell KO mice, respectively, stimulated with glucose and KCl. **d** Perifusion assay. Left panel, western blotting showing CASK expression levels in control and CASK-KO islets. Right panel, representative data (out of three experiments) showing that CASK-KO islets had impaired insulin secretion in the perifusion assay. **e** (i) Representative images from transmission electron micrograph showing the distribution of dense core vesicles in the control or CASK–depleted (CASK Ri-1) beta cells after glucose stimulation. An orange line was used to outline the cell membrane. Distances from yellow, purple and pink lines to cell membrane were 25 nm, 80 nm and 160 nm, respectively. Arrows and arrowheads indicate the vesicles located within 160 nm from the cell membrane and vesicles pinching off from the cell surface, respectively. (ii) Quantification of vesicle distribution after stimulation and total vesicle number in unstimulated cells were shown in right panels. (iii) In total, 13 samples of control islets and 18 samples of CASK–depleted islets were analyzed. **f** Insulin vesicle fusion induced by glucose/KCl in INS-1E cells. (i) bright-field image of (ii). (ii) Maximum projections from a representative experiment recorded by TIRFM showing release events over the basal period (10 mins) and first 10 mins of both the glucose and KCl stimulations. Scale bar, 50 μM. (iii) average number of release events per minute per field of view detected in control (NC) and CASK RNAi cells. (iv) average event magnitude (Area X Event Intensity) per minute per field of view. Data are mean ± SEM of 4 independent experiments per condition with one field of confluent cells per experiment (~200 cells per field).

CASK was ubiquitously expressed in various mouse tissue types, with the highest expression level detected in the brain and islets (clearly in insulin-secreting β cells) (Supplementary Fig. S1a, b). CASK knockdown in INS-1E cells (a rat pancreatic β cell line) strongly suppressed both glucose-stimulated and KCl-induced insulin secretion (Fig. 1a), while expression of the “siRNA-resistant” forms of CASK restored glucose-stimulated insulin secretion to a normal level (Fig. 1b), demonstrating the functional specificity of CASK in regulating insulin secretion.

To evaluate the physiological relevance of CASK in pancreatic β cells, islets were isolated from the pancreases of *CASK*-knockout and control mice, and insulin secretion was assessed. Depletion of CASK in mouse islets resulted in significantly decreased insulin secretion upon stimulation with either glucose or KCl in both the static and dynamic secretion assays (Fig. 1c, d) compared to that from control islets isolated from RIP-Cre mice. This effect was not due to alteration of the islet size or the β cell mass (Supplementary Fig. S2a) and indicated a direct role of CASK in insulin secretion in vivo.

Since CASK knockdown did not alter the total insulin content in INS-1E cells or islets (Supplementary Fig. S2b), the reduction in insulin secretion was unlikely to be caused by decreased insulin production. Depletion of CASK inhibited both glucose-stimulated and KCl-induced insulin secretion in β cells and islets, suggesting that CASK is most likely a regulator of the final steps in insulin vesicle secretion. We examined the secretory granules in CASK-knockdown β cells using electron microscopy and found that the distribution of secretory granules in β cells differed significantly between control and CASK-depleted cells, although the total vesicle number and general size remained the same (Fig. 1e i, iii). Fewer vesicles were observed in the vicinity of the plasma membrane in CASK-knockdown cells than in control cells. Under glucose stimulation, vesicles closely attached to the cell surface were observed frequently in control cells but seldom in CASK-knockdown cells (Fig. 1e i, ii). These findings indicate that CASK directly affects insulin vesicle docking.

TIRFM was employed to further investigate the role of CASK in regulating the fusion of insulin-containing vesicles. Individual insulin release events were monitored using the nonpermeable fluorescent zinc sensor FluoZin-3, which binds to Zn^2+^ cosecreted with insulin from INS-1E cells^13^. Vesicle fusion events occurred less frequently in CASK-knockdown cells than in control cells in response to glucose or KCl stimulation (Fig. 1f i-ii, Supplementary Video S1). Interestingly, CASK knockdown also resulted in a reduction in the amount of zinc released per event (event magnitude = corrected intensity × area) (Fig. 1f iii). This reduction is consistent with a reduction in the size and/or opening time of the fusion pore, suggesting fewer full-fusion and more kiss-and-run type events (Fig. 1f iv). The largest effects of CASK knockdown were observed in the first phase of glucose-stimulated release (the first 10 min after glucose stimulation) and upon KCl-mediated secretion, like the data obtained in the perifusion assay (Fig. 1d). These data demonstrate that CASK plays a prominent role in regulating the insulin vesicle fusion mode and frequency.

### CASK forms a functional protein complex with Mint1 and Munc18-1 in a manner regulated by glucose stimulation

**Fig. 2:**
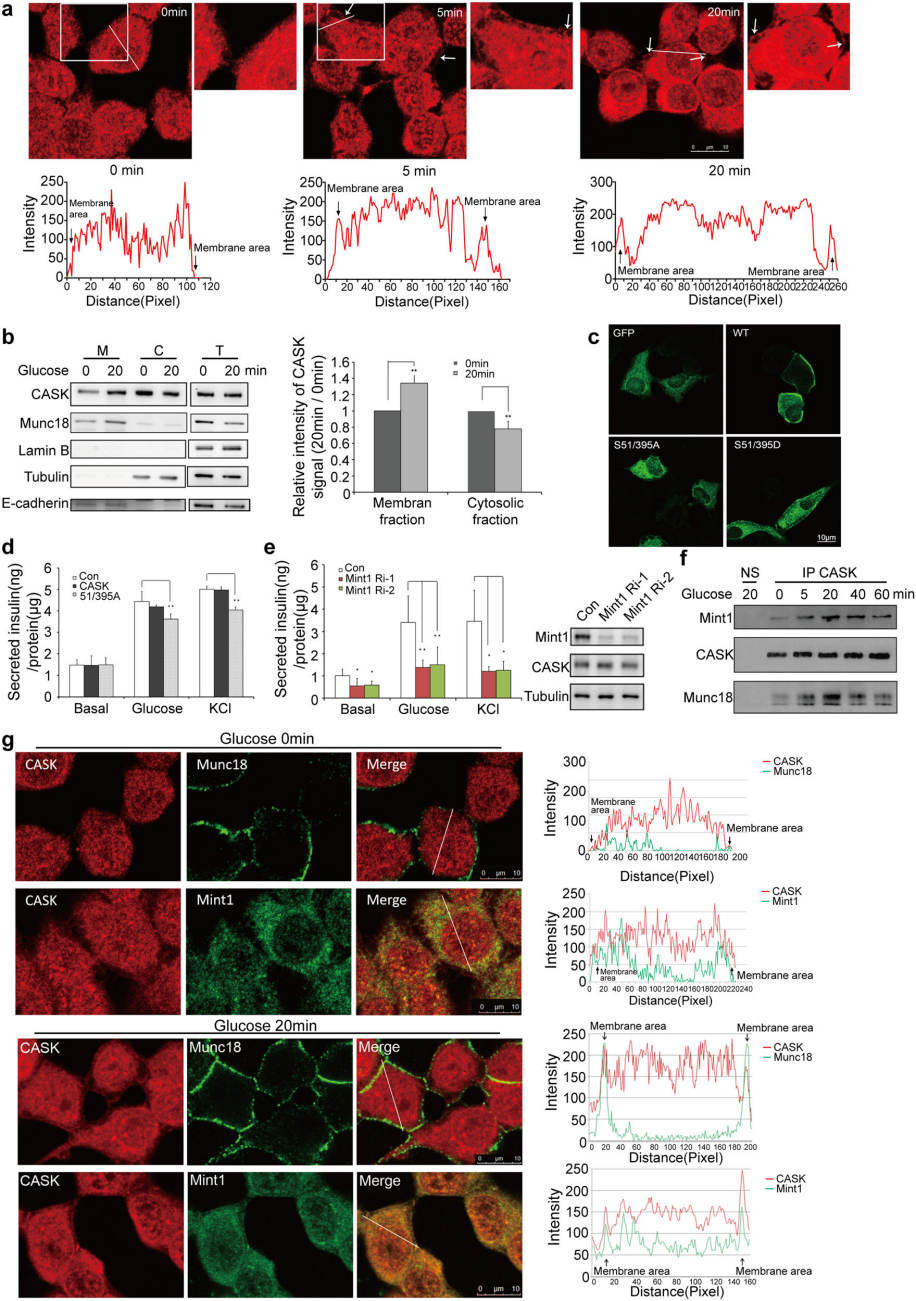
CASK formed a complex with mint1-munc18-1 upon glucose stimulation and regulated mint1-munc18 interaction. **a** Immunomicrographs reveal membrane translocation of CASK in INS-1E cells following glucose stimulation (upper panel). Represented peak curve diagrams showing cellular distribution of CASK immunofluorescent signals measured by Image J with linear scanning (lower panel). **b** Subfractionation assay for membrane translocation of CASK upon glucose stimulation showed by western blot analysis (left panel). Quantification of the signal of CASKusing a densitometer (right panel). M: membrane fraction; C: cytosolic fraction; T: total lysate. **c** Overexpressing CASK/WT, CASK/S51/395A, CASK/S51/395D mutants in INS-1E cells. Immunomicrographs showing the subcellular localization of different CASK mutants. **d** Histograms showing expression of CASK-S51/395A mutant inhibited insulin secretion. **e** The effect of Mint1 on insulin secretion in INS-1E cells (left panel). Right panel, western blotting showing the efficiency of Mint1 knockdown (two distinct siRNA fragments, Ri-1 and Ri-2). **f** Western blot analysis showed that glucose-induced increased co-immunoprecipitation of endogenous CASK, Mint1 and Munc18-1 in a time-dependent manner in INS-1E cells. Non-immune serum (NS) was used as a negative control for immunoprecipitation. **g** The subcellular distribution of CASK, Munc18-1 and Mint1 before or after glucose stimulation. Cells were stained with anti-CASK (red) and anti-Munc18-1 (green) or anti-Mint1 (green) (left panel). Image pro plus was used to analyze the co-localization of the two proteins. Arrows indicates the membrane region. Note the overlap of the red and green signal peaks at the cell membrane after glucose stimulation (right panel).

Consistent with a role for CASK in regulating vesicle docking and fusion near the cell membrane, glucose stimulation enhanced the membrane localization of CASK, as revealed by immunostaining (Fig. 2a) and cellular fractionation assays (Fig. 2b). Interestingly, glucose stimulation in INS-1E cells increased the membrane accumulation of CASK (Fig. 2a). Moreover, the membrane localization of CASK was itself important for promoting insulin secretion. A previous study reported that Cdk5 activation-mediated phosphorylation of CASK on Ser51 and Ser395 is necessary for its membrane localization^14^. The S51A/395A CASK mutant could not localize to the plasma membrane (Fig. 2c), and its expression inhibited glucose-stimulated insulin secretion from INS-1E cells (Fig. 2d), suggesting the involvement of CASK membrane localization in insulin secretion.

**Fig. 3:**
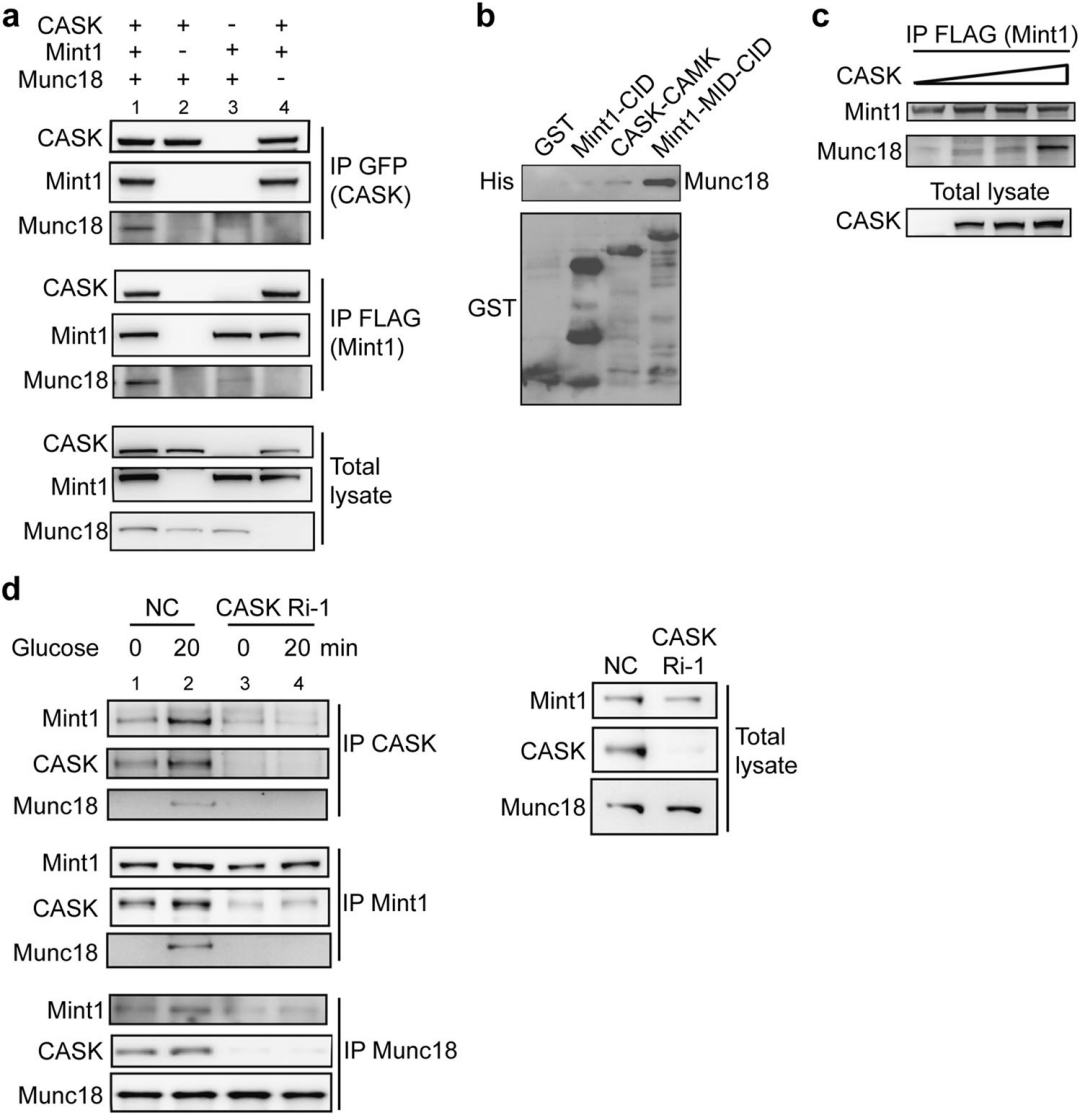
CASK-Mint1 interaction facilitates CASK/Mint1/Munc18-1 complex formation. **a** Co-immunoprecipitation of overexpressed CASK-GFP, Mint1-Flag and Munc18-1 in 293 cells showed by western blot analysis. Immunoprecipitations (IPs) of CASK and Mint1 were carried out with anti-GFP or anti-FLAG ABs., respectively. **b** Munc18-1 bound to Mint1-MID-CID directly, but not to CASK-CaMK. Recombinant his-Munc18-1 was incubated with different fusion proteins of GST-CaMK (CASK), CST-Mint1-CID and GST-Mint1-MID-CID (lower panel) as indicated. Upper panel, his-Munc18-1 detected by western blot with anti-his AB. Lower panel, different GST-fusion proteins detected by WB with anti-GST AB. **c** Co-immunoprecipitation of exogenous CASK, Mint1-Flag and Munc18-1 with anti-flag antibodies when CASK was expressed at different levels in 293 cells. For **a** and **c**, anti-tag antibodies were used to detect the proteins co-immunoprecipitated with CASK or Mint1. **d** The effect of CASK-depletion on the formation of CASK/Mint1/Munc18-1 tripartite complex in native INS-1E cells stimulated with glucose. Left panel, western bolt analysis for detection of the formation of CASK/Mint1/Munc18-1 complex precipitated by IPs with CASK, Mint1 and Munc18 antibodies. Right panel, expression of CASK, Mint1 and Munc18 revealed by western bolt analysis. Ri-1: CASK-specific siRNA; NC: non-silencing control siRNA.

Next, we sought to identify the components of the secretion machinery possibly regulated by CASK. Loss or overexpression of CASK did not appreciably affect the expression of Mint1, Munc18-1, Syntaxin-1, VAMP2 or SNAP25 (Supplementary Fig. S3). We further evaluated Mint1 on the basis of previous work showing that neuronal CASK often functions cooperatively with Mint1^10^. Similar to knockdown of CASK, knockdown of Mint1 resulted in a dramatic decrease in insulin secretion after glucose or KCl stimulation (Fig. 2e), indicating that Mint1 is critical for insulin secretion. Mint1 was identified as the interacting protein of the SM protein Munc18-1, a key regulator of the SNARE complex^15^. Coimmunoprecipitation experiments in wild-type INS-1E cells showed that CASK associated with Mint1 and Munc18-1. Interestingly, the interaction between these proteins was notably promoted by glucose stimulation in β cells (Figs. 2f and 3d). Consistent with this finding, immunofluorescence staining analysis revealed colocalization of CASK with Mint1 and Munc18-1 on the plasma membrane of INS-1E cells upon glucose stimulation (Fig. 2g). These data suggest that CASK might form a functional protein complex with Mint1 and Munc18-1.

### CASK-Mint1 binding facilitates the formation of the CASK/Mint1/Munc18-1 ternary complex

A previous study suggested that Mint1 interacts with the CaMK domain of CASK via its CID and with Munc18 via its Munc18-interaction domain (MID)^10^. However, whether and how these three proteins directly form a ternary complex are incompletely clarified. To address these questions, we performed coimmunoprecipitation assays with CASK, Mint1 and Munc18-1 in 293 cells. These three proteins formed a physiological complex when they were co-overexpressed (Fig. 3a, lane 1). CASK bound to Mint1 in either the presence or absence of Munc18-1 (Fig. 3a, lanes 1 and 4). Unexpectedly, CASK did not coprecipitate with Munc18-1 in the absence of Mint1 (Fig. 3a, lane 2 in the upper panel). More interestingly, a significant decrease in the Mint1-Munc18-1 interaction was found in the absence of CASK (Fig. 3a, lane 3 in the middle panel). These results imply two possibilities: (1) CASK does not bind to Munc18-1 directly, and Mint1 is a mediator bridging CASK and Munc18-1, and (2) binding of CASK to Mint1 regulates the interaction between Mint1 and Munc18-1. Additional experiments were performed to further investigate these two possibilities. In vitro pulldown binding assays revealed that Munc18-1 bound the Mint1-MID but not Mint1-CID, CASK-CaMK (Fig. 3b) and other domains of CASK (data not shown). In addition, increasing the CASK expression level enhanced the Mint1-Munc18-1 interaction in a dose-dependent manner, as shown in Fig. 3c. Moreover, CASK knockdown appreciably weakened the interaction between Mint1 and Munc18-1 induced by glucose stimulation, suggesting a functional role of CASK in the Mint1-Munc18 interaction (Fig. 3d). These findings suggest that CASK and its binding to Mint1 are required for modulating ternary complex formation, which is clearly regulated by glucose.

To further emphasize the association of the CASK-Mint1 interaction with insulin secretion, rescue experiments were performed in INS-1E cells. CASK overexpression did not rescue the inhibitory effect of Mint1 depletion on insulin secretion, and Mint1 overexpression did not rescue the inhibitory effect of CASK depletion on insulin secretion (Supplementary Fig. S4a, b), suggesting that CASK and Mint1 must cooperate to regulate insulin secretion. Loss of either component results in disruption of the complex and decreases vesicle secretion. Consistent with this idea, exogenous expression of a Mint1 mutant lacking the CASK interaction domain (Mint1/∆CID) but still able to bind Munc18 inhibited insulin secretion from INS-1E cells (Supplementary Fig. S4c). Taken together, these results demonstrate that CASK-driven formation of a ternary complex with Mint1 and Munc18-1 may have physiological significance in insulin secretion.

### Mint1 wraps around CASK-CaMK like a “whip” in the CASK/Mint1 complex

**Fig. 4:**
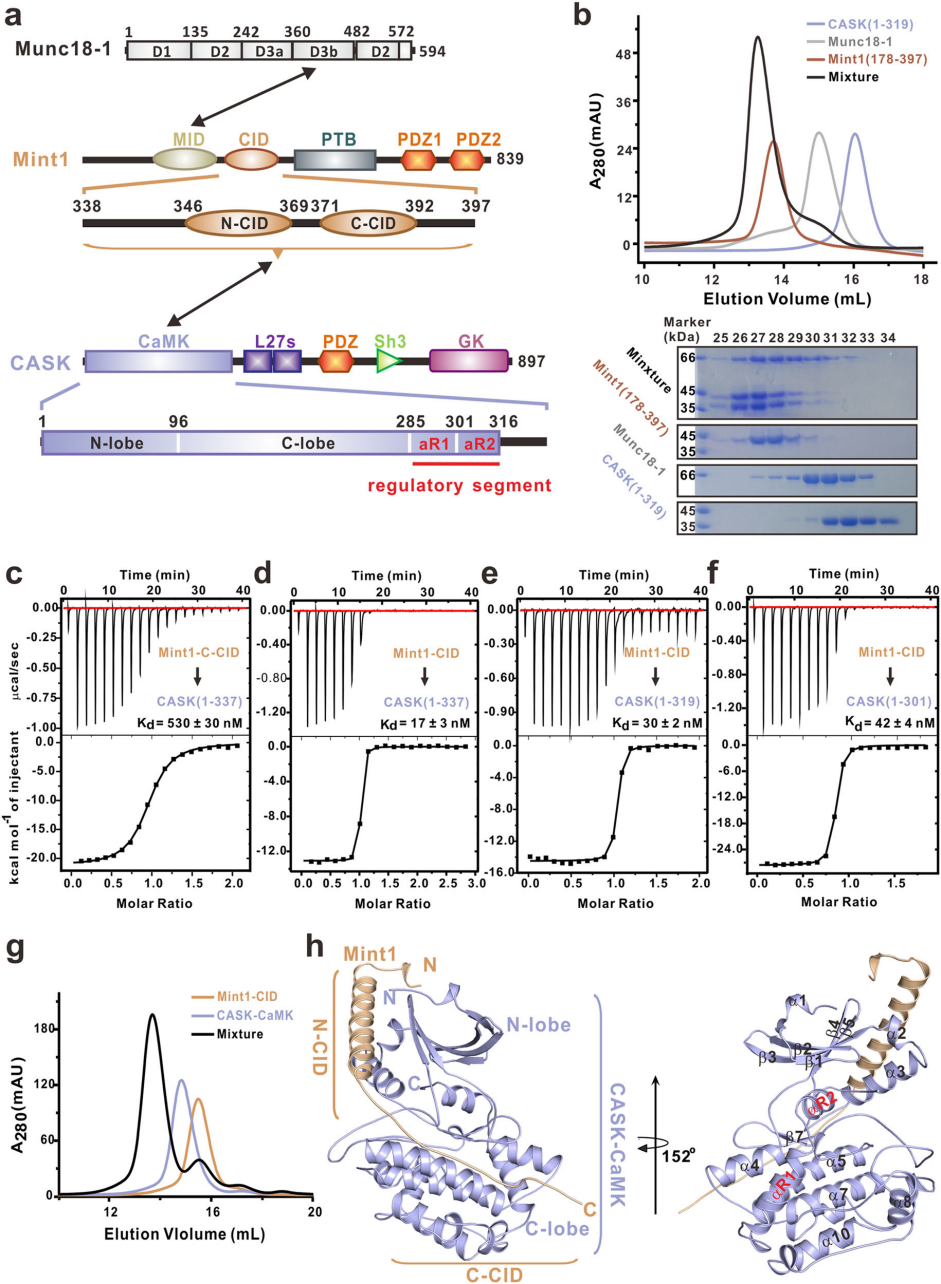
Unique CASK-Mint1 interaction. **a** Schematic diagram of the domain organization of Munc18-1, Mint1 and CASK. The figure illustrates the detailed boundaries of Mint1-CID and CASK-CaMK. The color coding of the domains is used throughout the entire manuscript except as otherwise indicated. **b** Analytical gel filtration chromatography showing the CASK/Mint1/Munc18-1 complex formation. The eluted peak fractions of OD_280_ were further analyzed by SDS-PAGE followed by Coomassie Brilliant Blue staining. The fraction numbers and molecular mass markers are indicated on the top and the left of SDS-PAGE gels. **c**–**f** Interactions between various forms of CASK-CaMK and Mint1-CID measured by ITC assay, including Mint1-C-CID (residues 371-397) and CASK(residues 1-337) (**c**), Mint1-CID (residues 338-397) and CASK(residues 1-337) (**d**), Mint1-CID (residues 338-397) and CASK(residues 1-319) (**e**) and Mint1-CID (residues 338-397) and CASK (residues 1-301) (**f**). **g** Analytical gel filtration chromatography showing the stable CASK/Mint1 complex formation. **h** Ribbon diagram of the structure of the CASK/Mint1 complex. The structure is shown from two different side views with the same color coding in panel A. The secondary structures of CASK-CaMK and Mint1-CID are labeled.

Next, we sought to clarify whether the Mint1-MID-CID can bridge CASK and Munc18 in vitro. Via gel filtration chromatography with purified CASK-CaMK (residues 1–319), Mint1 (residues 178–397, including the CID and MID) and Munc18-1 (full length) proteins (Fig. 4a), formation of the ternary complex formation in vitro was verified, as shown in Fig. 4b. Since ternary complex formation depends on the CASK-Mint1 interaction, we sought to understand this interaction in detail by determining the crystal structure of the CASK/Mint1 heterodimer. We first confirmed that the interaction between CASK and Mint1 is mediated by CASK-CaMK and Mint1-CID. To evaluate the contribution of the N-terminal half of the CID (N-CID) to the interaction, we compared the binding of CASK-CaMK to the C-CID and the entire CID by isothermal titration calorimetry (ITC) (Fig. 4c, d). The C-CID (residues 371–397) bound to CASK-CaMK (residues 1–337) with a Kd of ~500 nΜ. Interestingly, the entire CID significantly enhanced the binding affinity by ~30-fold and formed a stable complex with CaMK in solution (Fig. 4g), indicating that the N-CID of Mint1 is also essential for its binding to CASK. These data demonstrate that the entire CID of Mint1, not just the C-CID, is required for stable CASK/Mint1 complex formation.

To elucidate the molecular basis of the CASK-Mint1 interaction, we next determined the structure of the CASK/Mint1 complex by X-ray crystallography. Since the entire CID of Mint1 is required for stable complex formation (Fig. 4c, d), we performed crystal screening of the CASK/Mint1 complex using the whole CID and different CASK-CaMK domain fragments with various C-terminal ends. After extensive assessment, we found that removal of the flexible tail (residues 320-337) of CASK-CaMK greatly improved the crystal quality. Moreover, removal of the flexible tail of CASK-CaMK had little impact on the binding of Mint1-CID (Fig. 4e, f). The structure was solved by the molecular replacement method and was finally refined to ~2.4 Å resolution (Supplementary Table S1). In the complex structure, CASK-CaMK adopts a typical protein kinase fold (Fig. 4h). Surprisingly, the Mint1-CID peptide stretches like a “whip” wrapping around CASK-CaMK from the N-lobe to the C-lobe (Fig. 4h). The N-CID forms a rigid α-helix that packs into a groove formed by α2, α3 and β4 in the N-lobe, while the C-CID is rather flexible and anchors into a preformed pocket within the C-lobe. Thus, the crystal structure supports the biochemical data (Fig. 4c–f), suggesting that both the N-CID and C-CID mediate the interaction between Mint1 and CASK-CaMK.

### CASK-CaMK recognizes Mint1 via two unique hydrophobic pockets

**Fig. 5:**
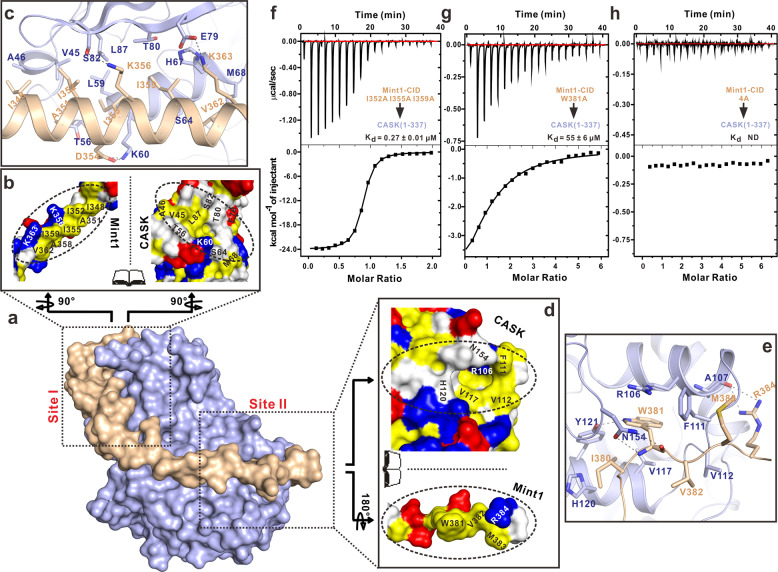
Interaction interface in the CASK/Mint1 complex. **a** A surface representation of the CASK/Mint1 complex showing that Mint1-CID wraps around CASK-CaMK. The interaction interface between CASK and Mint1 can be divided into two sites (sites I and II, highlighted by dashed boxes). **b**, **d** An “Open-book” view of the two interaction sites of the CASK/Mint1 complex by a surface representation. Site I is contributed by the helical packing between the N-lobe of CASK-CaMK and the N-CID of Mint1 (**b**), and site II is mainly contributed by the interaction interface between the C-lobe of CASK-CaMK and the C-CID of Mint1 (**d**). In the drawing, the hydrophobic, positively charged, negatively charged residues and remaining residues are colored in yellow, blue, red and white, respectively. **c**, **e** A combined ribbon-and-stick model showing the interface in site I (**c**) and site II (**e**). The side chains of the key residues involved in the interface packing are shown as sticks. **f**–**h** Interaction between CASK-CaMK (residues 1–337) and Mint1-CID (residues 338-397) mutants measured by ITC assay: (**f**) I352A I355A I359A; (**g**) W381A and (**h**) 4A.

The structure of the CASK/Mint1 complex showed that the interaction interface between CASK-CaMK and Mint1-CID (~1450 Å^2^) is largely mediated by hydrophobic packing and can be further divided into two sites, I and II (Fig. 5a). At site I, where the N-lobe interacts with the N-CID, the hydrophobic side of the N-CID helix formed by I348, I352, I355, I359 and V362 packs into the hydrophobic groove constructed by V45, A46, H67, M68 and L87 from the N-lobe (Fig. 5b, c). These interactions are further stabilized by the electrostatic and hydrogen bonding interactions between D345, K356 and K363 in the N-CID helix and K60, S82 and E79 in the N-lobe (Fig. 5b, c). At site II, where the C-lobe interacts with the C-CID, I380, W381, V382 and M383 in the C-CID anchor into a deep hydrophobic pocket formed by A107, F111, V112, V117 and Y121 in the C-lobe (Fig. 5d, e). The R106-W381 cation-π pair, together with 5 hydrogen bonds (Asn^154^_CaMK_-Trp^381^_Mint1_, Tyr^121^_CaMK_-Trp^381^_Mint1_ and Ala^107^_CaMK_-Arg^384^_Mint1_) (Fig. 5d, e), further locks the entire Trp381 residue into the CaMK hydrophobic pocket. Taken together, these observations indicate that the extensive hydrophobic packing within sites I and II drives the specific formation of the stable CASK/Mint1 complex.

Guided by the CASK/Mint1 structure, we introduced several point mutations in Mint 1, I352A/I355A/I359A in the N-CID and W381A in the C-CID of Mint1, to identify the key residues in CASK-Mint1 binding. The mutations in the N-CID reduced the binding affinity by ~15-fold (to a level like that of the C-CID alone) (Fig. 5f). In contrast, the C-CID W381A mutation more significantly impacted the CASK-Mint1 interaction and dramatically decreased the binding affinity (increasing Kd from ~20 nΜ to ~55 μΜ) (Fig. 5g). Thus, interaction site II may be the primary binding site, while auxiliary site I may further enhance binding. As expected, combining the mutations in the N-CID and C-CID (quadruple 4A mutation, I352A/I355A/I359A/W381A) completely abolished the CASK-Mint1 interaction (Fig. 5h).

### The CASK-Mint1 interaction regulates Munc18-1 membrane localization and insulin secretion

**Fig. 6:**
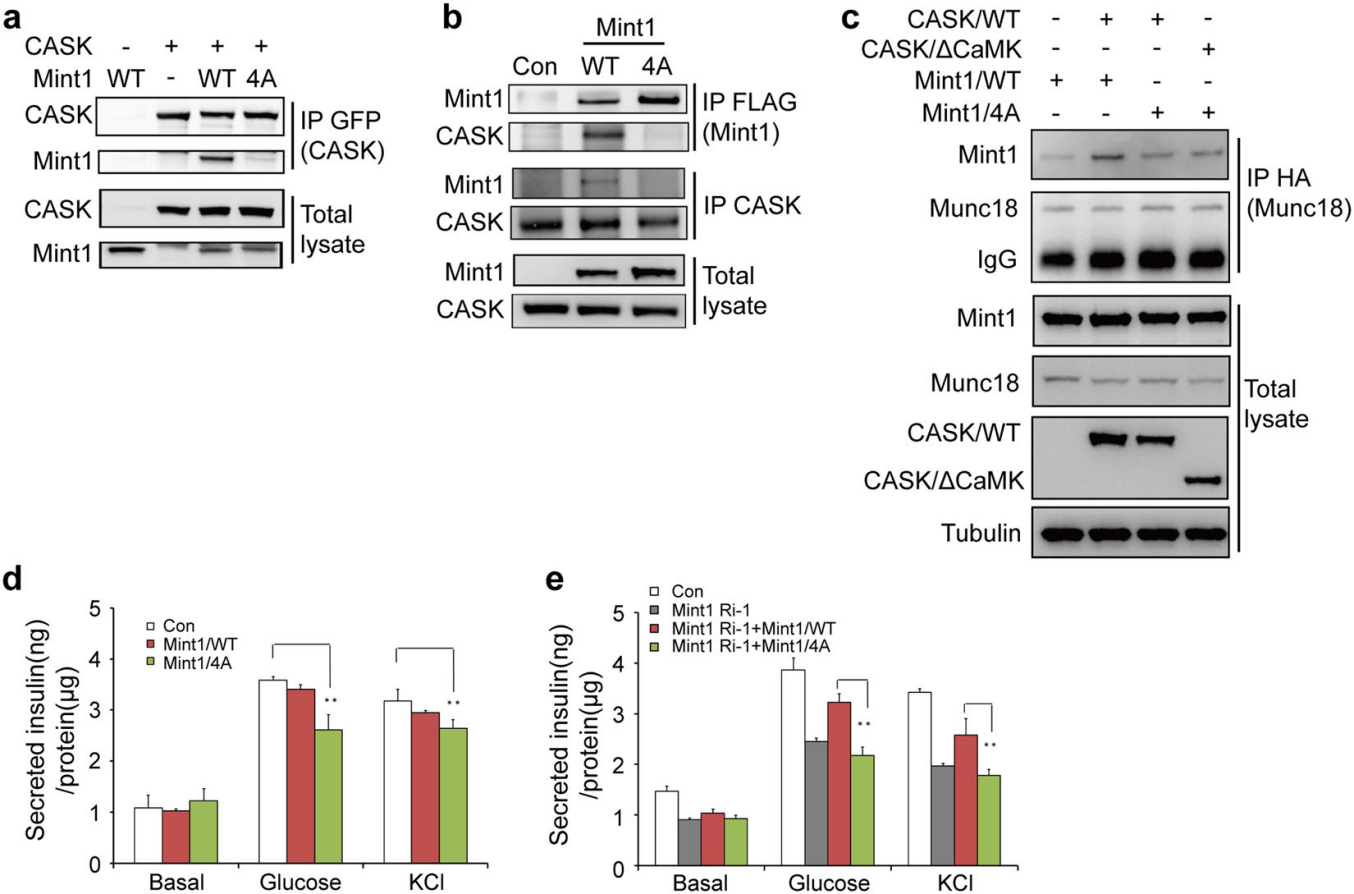
CASK-Mint1 interaction regulates insulin secretion. **a** Co-immunoprecipitation of exogenous CASK, Mint1 and Mint1/4A muntant in 293 cells. **b** Co-immunoprecipitation of CASK and Mint1 in glucose-stimulated INS-1E cells that were transfected with empty vector (Con), Mint1/WT or Mint1/4A mutant. **c** Co-immunoprecipitation of exogenous Munc18-1 and CASK in 293 cells. Mint1/WT or Mint1/4A mutant, CASK/WT or △CaMK mutant and Munc18-1 were overexpressed in 293 cells as indicated. **d** Overexpression of Mint1/4A mutant inhibited insulin secretion in INS-1E cells. **e** Reduced insulin secretion due to RNAi targeting Mint1 (Ri-1) could be rescued by siRNA-resistant wild type Mint1 (Ri-1+WT) but not the mutant (Ri-1 + 4A).

Consistent with the results of binding studies with recombinant proteins, full-length Mint1 carrying the quadruple 4A mutation (Mint1/4A) showed no detectable binding to exogenous CASK in 293 cells (Fig. 6a, lane 4) or to endogenous CASK in INS-1E cells (Fig. 6b, lane 3). Overexpression of CASK/WT promoted the interaction between wild-type Mint1 (Mint1/WT) and Munc18-1 (Fig. 6c, lane 2) but not between the Mint1/4A mutant and Munc18-1, even though Mint1/4A still interacted with Munc18-1 (Fig. 6c, lane 3). This pattern further supports the hypothesis that the interaction between Mint1 and CASK is important for regulating the binding of Mint1 to Munc18-1. As expected, overexpression of Mint1/4A in INS-1E cells inhibited glucose-stimulated and KCl-mediated insulin secretion (Fig. 6d), demonstrating that disruption of the CASK-Mint1 interaction impaired insulin secretion. We further compared the rescue effects of Mint1/WT and its mutants on insulin secretion. As expected, overexpression of Mint1/WT, but not the Mint1/4A mutant restored the insulin secretion level (Fig. 6e). Thus, collectively, these mutagenesis data demonstrate that the CASK-Mint1 interaction indeed plays an important role in insulin secretion.

**Fig. 7:**
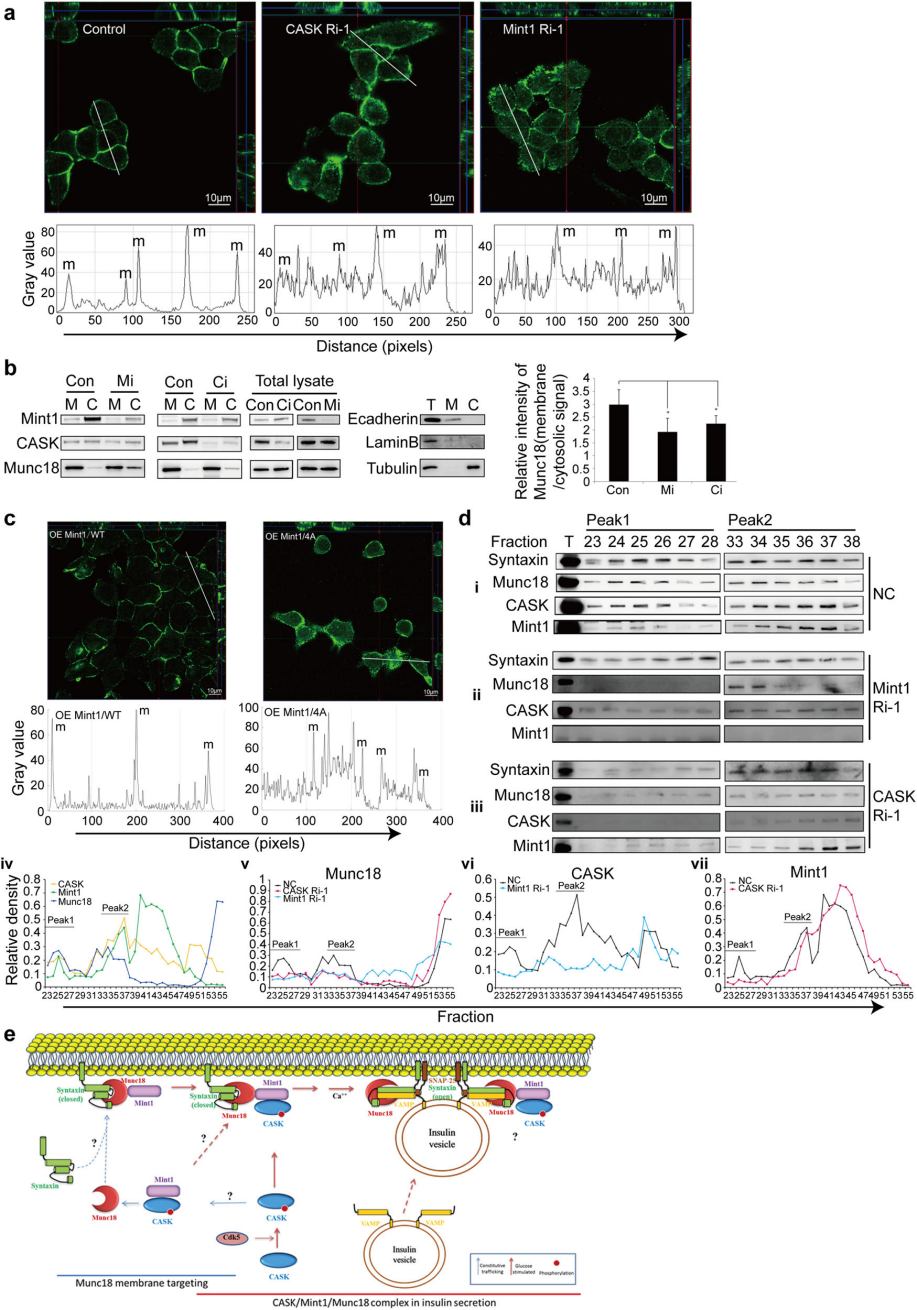
CASK-Mint1 interaction regulates Munc18-1 membrane localization. **a** Loss of CASK (CASK Ri-1) or Mint1 (Mint1 Ri-1) reduced the membrane localization of Munc18-1 which was detected by immunofluorescence using anti-Munc18-1 (green) (upper panel). Represented peak curve diagrams showing cellular distribution on membrane (m) and in cytoplasm of Munc18-1 immunofluorescent signals measured by Image J with linear scanning (lower panel). **b** Subcellular fractionation confirmed the increase of Munc18-1 in cytosolic fraction. and decrease in membrane fraction (M) when CASK or Mint1 was silenced (Ci & Mi). Con, control; T, total lysate. Ecadherin, LaminB and Tubulin were used as markers for membrane, nucleus and cytosol respectively (middle panel). The signals of Munc18-1 were quantified using a densitometer (right panel). **c** Overexpressing Mint1 4A mutant but not wild type (WT) Mint1 disrupted the membrane localization of Munc18-1 which was detected by immunofluorescence using anti-Munc18-1 (green) (upper panel). Represented peak curve diagrams showing cellular distribution on membrane (m) and in cytoplasma of Munc18-1 immunofluorescent signals measured by Image J. with linear scanning (low panel). *For **a** and **c** cells were treated with glucose. *For **a** and **c**, Z-projection in the X–Z direction and in the Y–Z direction are shown. The green and red lines indicate the orthogonal planes of the X–Z and Y–Z projection, respectively. *Note the different distribution pattern of Munc18a in different situations showed by the low panels of **a** and **c**. **d** Fractionation assays by gel filtration chromatography revealed co-fractionation of CASK, Mint1 and Munc18 in INS-1E cells transfected with non-silencing control siRNA (NC), CASK-specific siRNA (CASK Ri-1) or Mint1-specific siRNA (Mint1 Ri-1). After being stimulated with glucose, the lysate was fractionated by gel filtration chromatography using a Superose 6 10/300 GL column. The western blot images were showed for the representative fractions of Paek1 and Peak2 from INS-1E cell transfected with NC (i), Mint1 Ri-1(ii) and CASK Ri-1(iii). Eluted protein profiles from each fraction were quantified and normalized to T (total protein) for CASK, Mint1 and Munc18 (iv-vii) in INS-1E cells transfected with NC, CASK Ri-1 or Mint1 Ri-1, respectively. **e** The hypothetical model.

Membrane localization of Munc18-1 is essential for performing its tasks in vesicle release^4^. The correct localization of Munc18-1 is critical for syntaxin membrane localization and neurotransmitter secretion^16^. Thus, we sought to determine whether the CASK-Mint1 interaction mediates the distribution of Munc18-1. Immunofluorescence analysis in glucose-stimulated cells demonstrated that downregulation of either CASK or Mint1 in INS-1E cells relocalized Munc18-1 away from the membrane and into the cytosol (Fig. 7a). Similar results were obtained in the subcellular fractionation assay (Fig. 7b). More importantly, overexpression of the 4A mutant, but not Mint1/WT also altered the cellular localization of Munc18-1 (Fig. 7c), suggesting that the CASK-Mint1 interaction is important for Munc18-1 membrane localization. To further determine the cellular distribution of the CASK/Mint1/Munc18-1 complex and the functional relevance of this complex in glucose-induced insulin secretion, the protein complex fractions were analyzed by gel filtration chromatography. Compared to the unstimulated condition (data not shown), glucose treatment clearly promoted co-fractionation of CASK, Mint1 and Munc18-1, as well as Syntaxin. These proteins were obviously clustered in the first two major peaks, as shown in Fig. 7d I and iv. This co-distribution was lost in the absence of Mint1 (Fig. 7d ii) or CASK (Fig. 7d iii). Knockdown of CASK resulted in weakening of the first two major peaks of Munc18-1, Mint1 and syntaxin (Fig. 7d iii, v and vii). A similar effect was observed when Mint1 was depleted (Fig. 7d ii, v and vi). These data confirm that the distribution of the CASK/Mint1/Munc18-1 complex is glucose stimulation-related and that CASK/Mint1 can regulate the distribution of the ternary protein complex and its co-distribution with syntaxin.

## Discussion

In this study, we demonstrate a direct role of CASK in insulin vesicle docking and fusion via its interaction with Mint1-Munc18-1 and further explored the underlying molecular mechanism. Our study of the CASK/Mint1 complex crystal structure suggests that Mint1 is a unique binding partner of CASK. Upon glucose stimulation, CASK is translocated to the membrane and actively orchestrates CASK/Mint1/Munc18-1 complex formation, which is important for Munc18-1 localization and insulin secretion. Our work provides advanced evidence implicating the CASK/Mint1/Munc18-1 complex in insulin vesicle exocytosis. These findings extend the regulatory role of CASK in vesicle secretion from neurons to other types of secretory cells.

The results in INS-1E cells and the ex vivo data from the β cell-specific CASK-knockout islets (impaired insulin secretion) clearly show the physiological importance of CASK in insulin secretion. Since mice with β cell-specific CASK knockout are generated by crossing RIP-Cre and CASK floxed mice with overall knockdown of CASK expression in various tissues^17^, further investigation on the role of CASK in controlling glucose metabolism in vivo is limited. Thus, to prove CASK’s role in glucose metabolism in vivo, we further analyzed CASK expression in diabetic islets in several datasets from the Gene Expression Omnibus (GEO) and found that the expression levels of CASK are upregulated in db/db mice (9 weeks old, Supplementary Fig. S5a). Western blotting and immunofluorescence microscopy also demonstrated enhanced CASK expression in 8- to 10-week-old db/db mice (Supplementary Fig. S5b, c). Notably, Mint1 expression was also upregulated in these islets (Supplementary Fig. S5c). As 8- to 10-week-old db/db mice (in the early stage of diabetes) exhibit hyperinsulinemia^18^, we believe that this finding suggests compensatory upregulation of the CASK-Mint1 pathway in diabetic islets, which indicates a valid role of CASK-Mint1 in vivo.

### Mint1 is a unique binding partner for CASK

Our results suggest that Mint1 may be responsible for many, if not all, of the effects of CASK on insulin secretion. The CASK-Mint1 interaction is evolutionarily conserved^19^. In the brain, Mint1 is likely to be the predominant binding partner of CASK’s CaMK domain, which competes with Liprin (see below) to form a high-salt-resistant complex with CASK or binds to the CASK-neurexin complex simultaneously with Liprin^10,20^. In our study, loss of Mint1 inhibited insulin release, consistent with the findings of Waselle, who did not explore the underlying mechanism^21^. The glucose-stimulated CASK-Mint1 association and our further investigation with the Mint1 mutants supported the importance of the CASK-Mint1 interaction in insulin release. Our data clearly demonstrated that Mint1 is essential for ternary complex formation since Mint1 can bind simultaneously to Munc18-1 and CASK, as previously reported^8^, while CASK does not directly interact with Munc18-1. To the best of our knowledge, our study provides the first clear evidence supporting the presence of a CASK/Mint1/Munc18-1 regulatory axis in β cells.

**Fig. 8:**
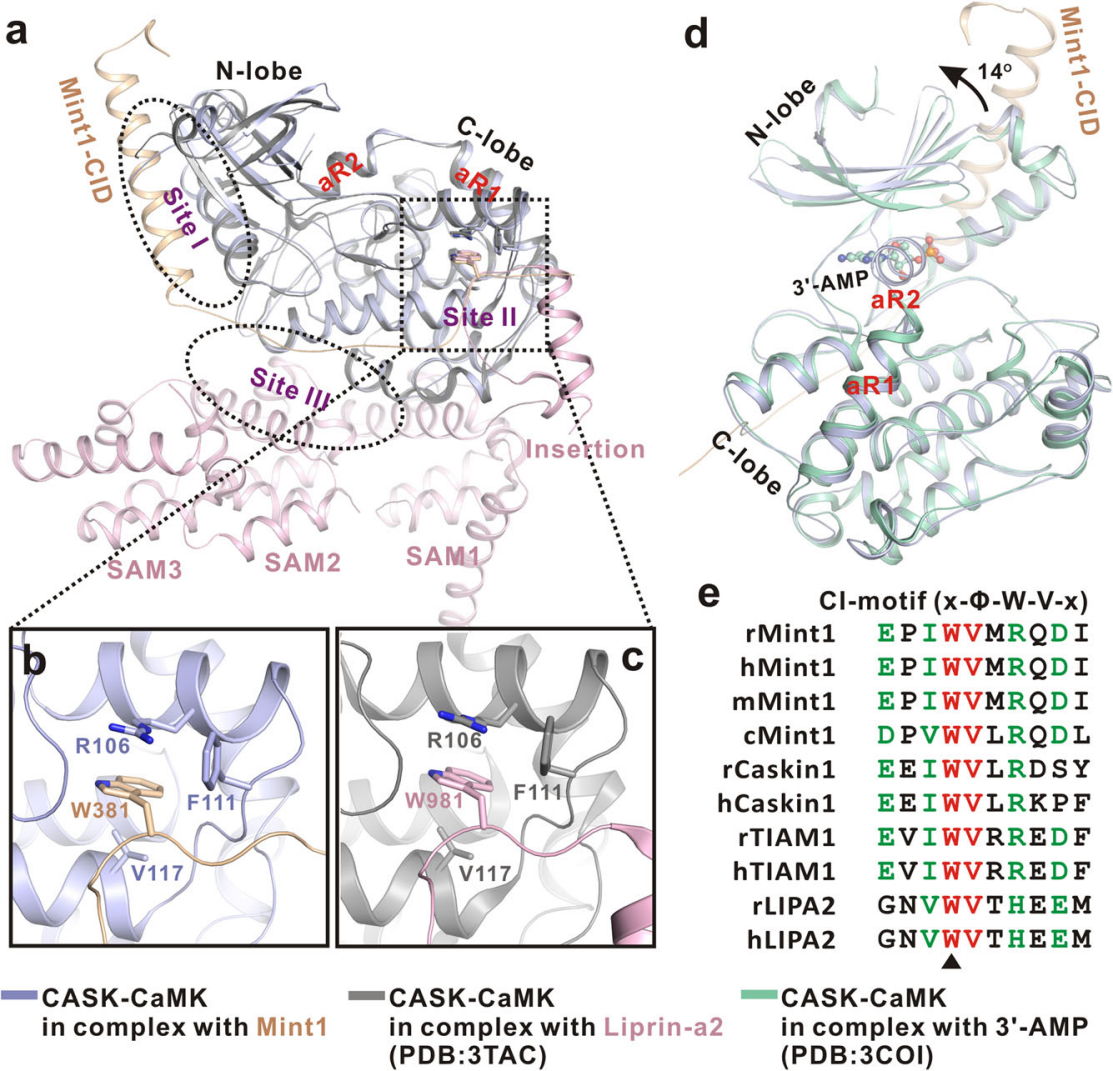
Structural comparison of CASK/Mint1 complex and other CASK complexes. **a** Overall structural comparison of CASK/Mint1 complex and CASK/Liprin-α2 complex. In the comparison, CASK-CaMK in the complex with Liprin-α2 is colored in dark gray and Liprin-α2 is colored in pink. **b**, **c** Similar Trp-binding pocket of CASK-CaMK in complex with Mint1 (**b**) and Liprin-α2 (**c**). **d** Structural comparison of CASK/Mint1 complex and AMP-bound CASK-CaMK. In the comparison, AMP-bound CASK-CaMK is colored in cyan with AMP-molecule shown as spheres. And ~14° orientation difference in their N-lobes of the two structures is indicated by an arrow. **e** Sequence alignment of the conserved “Trp”-containing CI-motifs from Mint1 and other CASK-binding partners.

To reveal the molecular details of CASK-Mint1 binding and verify its relevance, the crystal structure of the CASK/Mint1 complex was analyzed. Consistent with previous studies suggesting that the C-lobe of CASK-CaMK generally recognizes a linear Trp-containing sequence motif^22^, Liprin-α also contains a signature Trp residue within an insertion loop that can perfectly anchor into the preformed pocket in the C-lobe^23^ (Fig. 8a), reminiscent of the interaction between the Mint1 C-CID and the C-lobe. Since most binding partners of CASK-CaMK share this consensus sequence motif (Fig. 8e), they may competitively bind to this domain. Indeed, CASK-CaMK has been found to participate in alternative complexes in which the binding partners compete with each other^20^. Despite the similarity in the interaction, Liprin-α predominantly contacts the C-lobe (rather than both lobes) of CASK-CaMK (Fig. 8a–c), an interaction mode significantly different from that for the binding of CASK-CaMK to Mint1-CID. Thus, CASK-CaMK likely evolved to employ distinct additional sites (either the N-lobe or the back side of the C-lobe) to confer target-binding versatility for recognizing different targets. Moreover, the above structural difference in these two complexes might also be responsible for the competitive binding transition between them. The structural comparison could further suggest two conclusions. (1) CASK may need to select its binding partners according to different modes of biological simulation because it cannot bind to them simultaneously. Thus, CASK is an active organizer rather than a mere scaffold. Considering the enhanced membrane localization of CASK upon glucose stimulation, changes in the location of these molecules may contribute to this active selection process. (2) The unique binding mode of Mint1 to CASK implies a unique connection between these partners. To date, Mint1 has the highest binding affinity (Kd ~17 nΜ vs Kd~ 640 nΜ for liprin-α2_LH) among proteins to which CASK-CaMK binds^23^. This observation suggests that Mint1 is a major binding partner of CASK. The biological relevance of this idea is indicated by the glucose-enhanced CASK-Mint1 interaction and the impairment of insulin secretion when this interaction was disrupted in β cells. Our data support the hypothesis that CASK regulates insulin secretion mainly by interacting with Mint1.

Structural evaluation of the complex might shed light on whether and how CASK and Mint1 regulate each other. Regarding CASK, similar to the Liprin/CaMK complex structure, the last αR2 helix inserts directly into the ATP-binding cleft of CaMK (Fig. 8a, d), which induces opening of the cleft (with its N-lobe rotating counterclockwise by ~14° with respect to its orientation in the AMP-bound CASK-CaMK structure (Fig. 8d)^24^ and prevents its binding to ATP. Thus, the CaMK domain of CASK most likely adopts an inactive open conformation in the structure of these complexes (Fig. 8). This observation may also suggest an intriguing possibility that the kinase activity of CASK is regulated at least partially by its binding partners, such as Mint1. Since CASK can phosphorylate neurexin to regulate the formation of an important protein complex at the presynapse^20^, its kinase activity may have a similar impact on insulin secretion.

### CASK/Mint1-regulated Munc18-1 membrane translocation is important for insulin secretion

Munc18 is indispensable for vesicle release in both neurons and β cells^2^. Although all three Munc18 isoforms can be coimmunoprecipitated with CASK, Munc18-1 exhibits the highest affinity for CASK. This observation is consistent with the greater role of CASK in the first phase than in the second phase of insulin secretion (Fig. 1d), as Munc18-1 is crucial for phase I secretion and Munc18-3 is important for phase II secretion^25,26^. Mint1 interacts with Munc18-1 and Neurexin through different domains, participating in regulating the membrane localization of Munc18-1^15^. The interaction between Mint1 and Munc18-1 is promoted by CASK, especially under glucose stimulation (Fig. 3). Moreover, in the absence of Mint1, CASK did not interact with Munc18-1 (Fig. 3a). Collectively, these results indicate that the CASK/Mint/Munc18-1 complex is regulated by glucose-mediated signaling in β cells.

Direct binding of Munc18-1 to the syntaxin/SNARE complex is crucial for SNARE-mediated membrane fusion. Thus, cytosolic Munc18-1 must be targeted to the cell membrane. While one study suggested that syntaxin-1 might recruit Munc18-1 to the membrane^27^, another work suggested that Munc18-1 recruits syntaxin1 to the membrane^28^. In our CASK-Mint1-Munc18-1 model, loss of either Mint1 or CASK diminished the membrane localization of Munc18-1, suggesting that CASK/Mint1 participated in regulating the membrane localization of Munc18-1 in β cells. CASK and Mint1 obviously must cooperate in targeting Munc18-1 to the cell membrane, since the CASK-unbound 4A mutant of Mint1 disrupted the distribution of Munc18-1. The CASK/Mint1 complex has been shown to target various proteins to the membrane not only by anchoring them when they arrive at the membrane but also by directing their active transport to the membrane, since Mint1 can bind to the kinesin protein KIF17^29,30^. Alternatively, Mint1 may play a role in the transition of the Munc18-1/syntaxin/SNARE complex by directly regulating a conformational change in Munc18-1 in β cells. Importantly, Mint1 did not compete with syntaxin for binding to Munc18-1 in INS-1E cells (data not shown), in contrast to what has been suggested in PC12 cells^31^. Moreover, Syntaxin cofractionated with the ternary complex. Future studies should examine these possible mechanisms in more detail specifically regarding β cells and insulin secretion.

### Hypothetical working model

The CASK-Mint1-Munc18-1 model does not necessarily rule out other pathways^31^. Additional functions of CASK remain to be further investigated. In our CASK-Mint1-Munc18-1 model (for the regulation of insulin secretion but with potential extrapolation to other secretory systems, Fig. 7e), exposure to an appropriate stimulator, such as glucose, promotes membrane recruitment of CASK, which subsequently drives CASK/Mint1/Munc18-1 complex formation and ultimately results in increased insulin secretion. In this model, CASK may act as 1) a sensor receiving signals, such as calcium binding and phosphorylation induced by physiological/pathological stimuli, to activate the CASK-related pathway; 2) a direct regulator of Mint1/Munc18-1/other relevant proteins by functioning as either a scaffolding protein or a potential kinase; or 3) an active organizer to selectively recruit VDCC/Mint1/Munc18/SNARE into one confined, small region. Mint1 acts as 1) a direct regulator for Munc18-1 membrane localization and, probably, its conformational change; 2) a mediator to connect CASK with Munc18-1/syntaxin and other key proteins; and 3) a potential substrate for CASK or a regulator of its kinase activity (note the inactive open conformation of CaMK in the CASK/Mint1 complex) (Fig. 8). Moreover, Munc18-1 could be the target protein (core components of secretory machinery) to be regulated. Although further studies are needed to complete and verify this model, we believe that our study provides an intriguing scenario in which the CASK/Mint1 complex recruits Munc18-1 to the plasma membrane to establish a distinctive secretory machine to regulate insulin secretion.

## Materials and methods

### Cell culture

INS-1E cells (kindly provided by Dr. C. B. Wollheim, Geneva, Switzerland) were cultured as described previously^32^. INS-1E and 293 cells were kept at 37 °C in a humidified atmosphere containing 5% CO_2_.

### Subfractionation

Cells were scraped into buffer containing 10 mM Tris-HCl, pH 7.2, 25 mM KCl, 10 mM NaCl, 1 mM MgCl_2_, 0.1 mM EDTA, 1 mM NaF, and protease inhibitors (Roche). The lysate was passed through a 25 G needle and centrifuged at 200 × *g*. The supernatant was centrifuged at 720 × g, then again at 10,000 × *g*. The supernatant was then centrifuged at 100,000 × *g* to get the cytosolic fraction (supernatant) and the membrane fraction (pellet). Unless otherwise stated, all chemicals used in these experiments were obtained from Sigma.

### Insulin secretion assay

Insulin released from islets in static incubations, from perifused islets, and insulin secreted by INS-1E cells at different conditions were assayed as described in Supplemental methods.

### Immunofluorescence microscopy

Cells were fixed with cold methanol. Samples of mouse pancreas tissue were fixed with 4% paraformaldehdye, and cryoprotected with 30% sucrose in PBS. Tissues were frozen at –80 °C and cut into 5-μm sections on a sliding microtome (Leica). Images were photographed using a fluorescent confocal microscope (Zeiss LSM710, Germany).

### Electron microscopy

Samples were fixed in 4% paraformaldehyde and 0.05% glutaraldehyde fixative mixture in 0.1 M phosphate buffer (pH 7.4). Afterwards, cells were postfixed with 4% paraformaldehyde and 1% osmium tetroxide. After dehydration through a series of increasing ethanol concentrations, samples were embedded in Epon and polymerized at 60 °C. Afterwards, samples were sliced to ultrathin sections, which were then mounted on copper grids for staining with uranyl acetate and lead citrate, and analyzed under an electron microscope (JEM-1230; JEOL, Akishima, Japan).

### Total internal reflection fluorescence microscopy (TIRFM)

CASK-targeted siRNA or non-silencing control siRNA transfected INS-1E cells were bathed in Chelex-100 (BioRad)-treated, zinc-free KRBH containing 4 μM FluoZin (ThermoFisher) inside an OKOlabs incubator that was maintained at 37 °C and mounted on a Nikon Ti-E microscope. Zn^2+^ released from the cells will react with FluoZin in the medium and results in the fluorescence enhancements. TIRFM was performed using a 60× NA 1.65 objective with a 488-nm laser introduced into the excitation light path through the LApps H-TIRF module (Nikon) angled to generate a thin evanescent wave of ¡­-90 nm. Fluorescence was detected using an Andor iXon3 888 EMCCD camera running at 30 Hz (exposure time ~33 ms). Background fluorescence was removed by subtracting a 100-frame running average projection. Two separate methodologies were compared to detect release events and found to give similar results. These were an in house software package “TIRF explorer” (10.1109/ICCVW.2009.5457651) and a simple analysis pipeline developed in Fiji^33^ and cell profiler (PMC3072555) using object detection. The speed of the release events precluded us measuring the event kinetics and so we opted for a simple metric of the event size or magnitude. This was defined as the event area multiplied by the event intensity identified from a maximum projection of the frames that contained the event.

### Protein expression and purification

cDNA encoding CASK-CaMK domain (1–301, 1–319 and 1–337), various Mint1 fragments (178–397, 338–379 and 371–397) and Munc18-1 (full length) were cloned into the modified pET-32M vector that contain an N-terminal Trx-His_6_ tag. Recombinant proteins were expressed in *E. coli* BL21 (DE3) host cells at 16 °C. The expressed proteins were purified by Ni^2+^-Sepharose affinity chromatography followed by a size-exclusion chromatography (Superdex-200 26/60, GE Healthcare), and the fusion tag was removed by protease-3C digestion. The tag-removed proteins were further separated by another round of size-exclusion chromatography. To prepare the CASK-Mint1 complex for crystallization, the recombinant Mint1 and CASK were co-purified using the same methods described above.

### Isothermal titration calorimetry (ITC) assay

ITC was carried out on a MicroCalorimeter ITC200 at 25 °C by injecting aliquots of different fragments and mutants of Mint1 into the stirred CASK-CaMK in the calorimeter cell as described in Supplemental methods. Each experiment was repeated three times. The data were analyzed using ORIGIN 8.0.

### Crystallization, data collection and structure determination

Crystals of the CASK/Mint1 complex were obtained by the sitting drop vapor diffusion method at 16 °C in 0.2 M KI, 0.1 M MES buffer (pH 6.5). Before diffraction, crystals were cryoprotected by crystallization solution containing 15% glycerol. The diffraction data were collected at the beam line BL17U of the Shanghai Synchrotron Radiation Facility with a wavelength of 0.979 Å at 100 K and were processed and scaled using HKL2000^34^. For details concerning the structural determination, see Supplemental methods.

### Accession number

Atomic coordinates and structure factors have been deposited in the Protein Data Bank with PDB ID codes 6KMH.

### Statistics

Statistical significance was determined using the Student’s t test for unpaired data. A value of *P* ≤ 0.05 was taken as significant. *P < 0.05, **P < 0.01. Values presented in the figures represent means ± SDs from at least 3 experiments unless described otherwise in figure legends.

## Supplementary information

Supplemental Information, Figs, Table and Methods

Supplementary Movie S1 TIRFM showing insulin release events in CASK-RNAi or control INS-1E cells during the basal period and glucose stimulation.

## Supplementary information

**Supplementary Information** accompanies the paper at (10.1038/s41421-020-00216-3).

## References

[1] Komatsu M, Takei M, Ishii H, Sato Y (2013). Glucose-stimulated insulin secretion: a newer perspective. J. Diabetes Investig..

[2] Gaisano HY (2017). Recent new insights into the role of SNARE and associated proteins in insulin granule exocytosis. Diabetes Obes. Metab..

[3] Sudhof TC, Rothman JE (2009). Membrane fusion: grappling with SNARE and SM proteins. Science.

[4] Rizo J, Xu J (2015). The synaptic vesicle release machinery. Annu Rev. Biophys..

[5] Rizo J (2018). Mechanism of neurotransmitter release coming into focus. Protein Sci..

[6] Stepien KP, Prinslow EA, Rizo J (2019). Munc18-1 is crucial to overcome the inhibition of synaptic vesicle fusion by alphaSNAP. Nat. Commun..

[7] Misura KM, Scheller RH, Weis WI (2000). Three-dimensional structure of the neuronal-Sec1-syntaxin 1a complex. Nature.

[8] Biederer T, Sudhof TC (2000). Mints as adaptors. Direct binding to neurexins and recruitment of munc18. J. Biol. Chem..

[9] Hsueh YP (2006). The role of the MAGUK protein CASK in neural development and synaptic function. Curr. Med. Chem..

[10] Butz S, Okamoto M, Sudhof TC (1998). A tripartite protein complex with the potential to couple synaptic vesicle exocytosis to cell adhesion in brain. Cell.

[11] Olsen O, Moore KA, Nicoll RA, Bredt DS (2006). Synaptic transmission regulated by a presynaptic MALS/Liprin-alpha protein complex. Curr. Opin. Cell Biol..

[12] Spafford JD (2003). Calcium channel structural determinants of synaptic transmission between identified invertebrate neurons. J. Biol. Chem..

[13] Gee KR, Zhou ZL, Qian WJ, Kennedy R (2002). Detection and imaging of zinc secretion from pancreatic beta-cells using a new fluorescent zinc indicator. J. Am. Chem. Soc..

[14] Samuels BA (2007). Cdk5 promotes synaptogenesis by regulating the subcellular distribution of the MAGUK family member CASK. Neuron.

[15] Okamoto M, Sudhof TC (1997). Mints, Munc18-interacting proteins in synaptic vesicle exocytosis. J. Biol. Chem..

[16] Han GA (2011). Munc18-1 domain-1 controls vesicle docking and secretion by interacting with syntaxin-1 and chaperoning it to the plasma membrane. Mol. Biol. cell.

[17] Atasoy D (2007). Deletion of CASK in mice is lethal and impairs synaptic function. Proc. Natl Acad. Sci. USA.

[18] Wang, I. M. et al. Systems analysis of eleven rodent disease models reveals an inflammatome signature and key drivers. *Mol. Syst. Biol.* **8**, 594 (2012). 10.1038/msb.2012.24 PMC3421440 22806142

[19] Mukherjee, K., Slawson, J. B., Christmann, B. L., & Griffith, L. C. Neuron-specific protein interactions of Drosophila CASK-beta are revealed by mass spectrometry. *Front. Mol. Neurosci.* **7**, 58 (2014). 10.3389/fnmol.2014.00058 PMC4075472 25071438

[20] LaConte LE (2016). CASK stabilizes neurexin and links it to liprin-alpha in a neuronal activity-dependent manner. Cell Mol. Life Sci..

[21] Waselle L (2005). Role of phosphoinositide signaling in the control of insulin exocytosis. Mol. Endocrinol..

[22] Stafford RL, Ear J, Knight MJ, Bowie JU (2011). The molecular basis of the Caskin1 and Mint1 interaction with CASK. J. Mol. Biol..

[23] Wei Z (2011). Liprin-mediated large signaling complex organization revealed by the liprin-alpha/CASK and liprin-alpha/liprin-beta complex structures. Mol. Cell.

[24] Mukherjee K (2008). CASK functions as a Mg2+-independent neurexin kinase. Cell.

[25] Oh E, Thurmond DC (2009). Munc18c depletion selectively impairs the sustained phase of insulin release. Diabetes.

[26] Oh E, Kalwat MA, Kim MJ, Verhage M, Thurmond DC (2012). Munc18-1 regulates first-phase insulin release by promoting granule docking to multiple syntaxin isoforms. J. Biol. Chem..

[27] Pertsinidis A (2013). Ultrahigh-resolution imaging reveals formation of neuronal SNARE/Munc18 complexes in situ. Proc. Natl Acad. Sci. USA.

[28] Han GYA (2011). Munc18-1 domain-1 controls vesicle docking and secretion by interacting with syntaxin-1 and chaperoning it to the plasma membrane. Mol. Biol. Cell.

[29] Maximov A, Bezprozvanny I (2002). Synaptic targeting of N-type calcium channels in hippocampal neurons. J. Neurosci..

[30] Setou M, Nakagawa T, Seog DH, Hirokawa N (2000). Kinesin superfamily motor protein KIF17 and mLin-10 in NMDA receptor - Containing vesicle transport. Science.

[31] Schutz D, Zilly F, Lang T, Jahn R, Bruns D (2005). A dual function for Munc-18 in exocytosis of PC12 cells. Eur. J. Neurosci..

[32] Merglen A (2004). Glucose sensitivity and metabolism-secretion coupling studied during two-year continuous culture in INS-1E insulinoma cells. Endocrinology.

[33] Schindelin J (2012). Fiji: an open-source platform for biological-image analysis. Nat. Methods.

[34] Otwinowski, Z. & Minor, W. Processing of X-ray diffraction data collected in oscillation mode. *Methods Enzymol*. **276**, 307–326 (1997). 10.1016/S0076-6879(97)76066-X 27754618

