# Supplementary material for: CASK modulates the assembly and function of the Mint1/Munc18-1 complex to regulate insulin secretion
Source: Cell Discov. 2020 Dec 15;6:92. doi: 10.1038/s41421-020-00216-3 (PMC7736295; doi:10.1038/s41421-020-00216-3)
Supplement: Supplementary file 1 — Supplemental Information, Figs, Table and Methods [file 41421_2020_216_MOESM1_ESM.pdf]

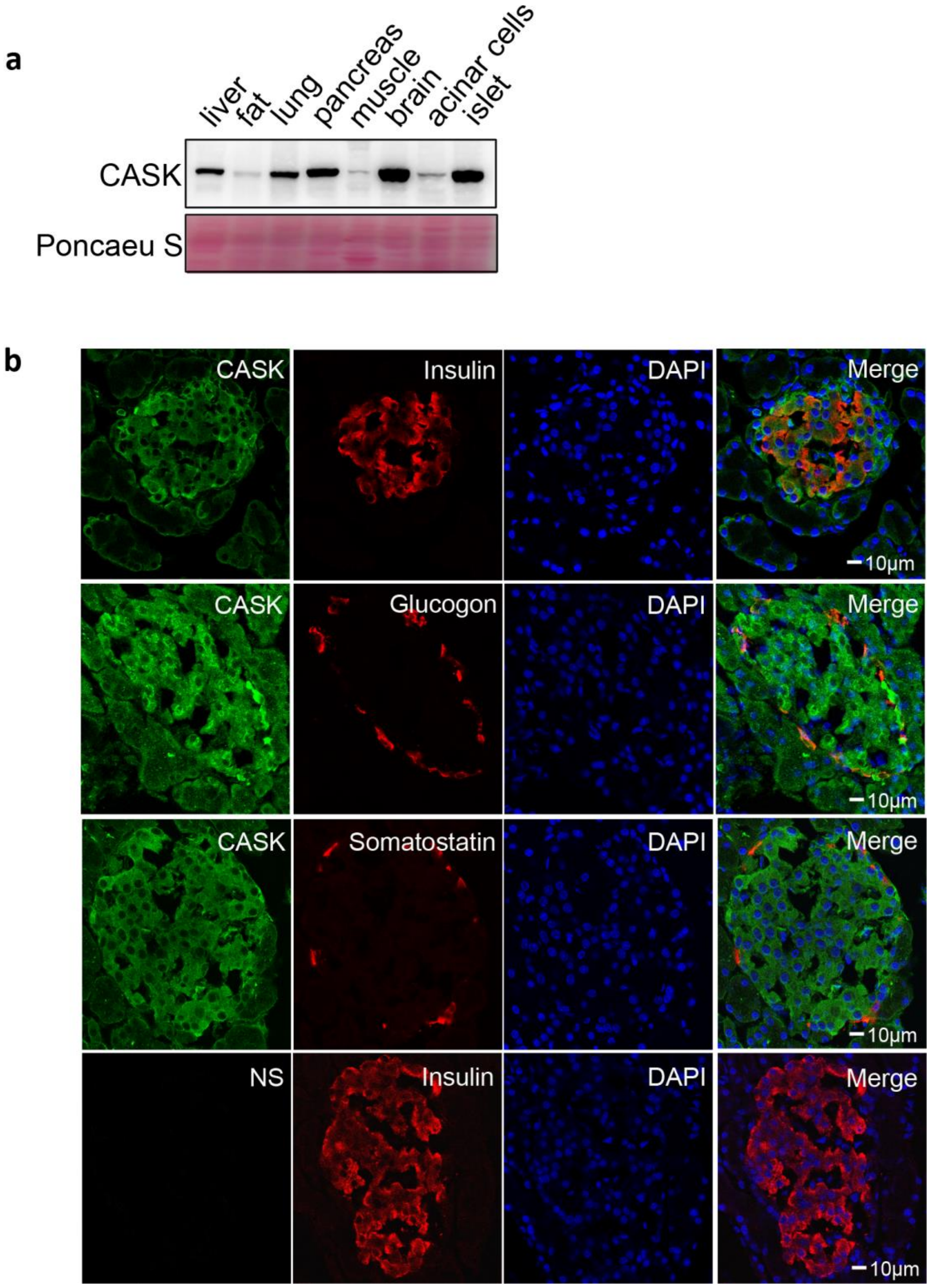

Supplementary Fig. S1 CASK expression in normal mice islets (a) Western blot analysis showing the expression of CASK in different mouse tissues, with Ponceau S staining as a loading control. (b) Immunofluorescence microscopy showing CASK expression in pancreas. Fixed tissue sections were stained with anti-CASK (green), anti-insulin (red, beta cells), anti-glucagon (red, alpha cells), anti-somatostatin (red, delta cells) and DAPI (DNA, blue). NS (non-immune sera) was used as a negative control.

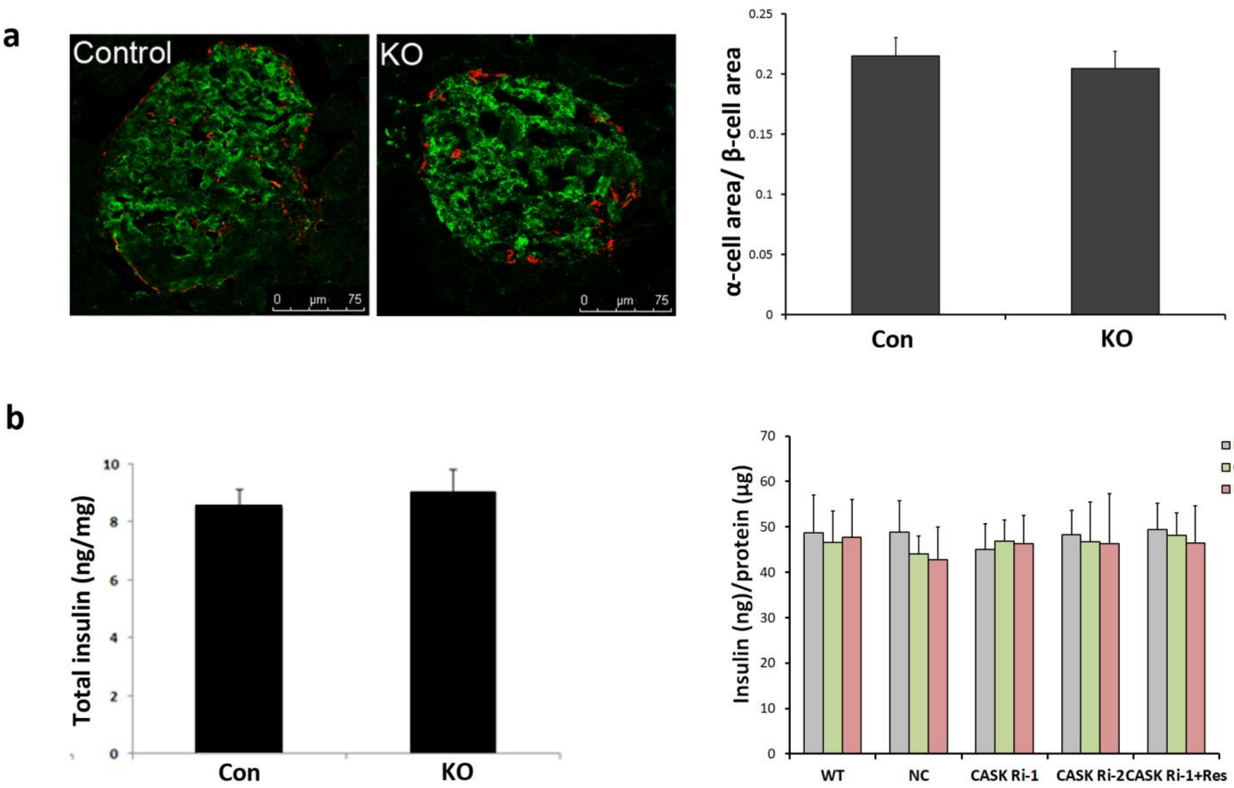

Supplementary Fig. S2 Characterization of CASK-knockout islets. (a) The architecture of islets from control and CASK knockout (KO) islets. Insulin (green) was used to indicate beta cells, glucagon (red) was used to indicate alpha cells. Left panel, Immunofluorescence analysis; right panel, quantification of cellular composition of the islets (total alpha cell area/total beta cell area). Data were collected from four mice each group. The distance between sections > 300 μm. (b)Total insulin content in islets (left panel) or INS-1E cells (right panel). Left panel, Data were collected from three islets each group. Right panel, INS-1E cells were treated as indicated. Statistical analysis revealed no significant difference between the data from control islet/cells and CASK knockout islet/siRNA treated cells.

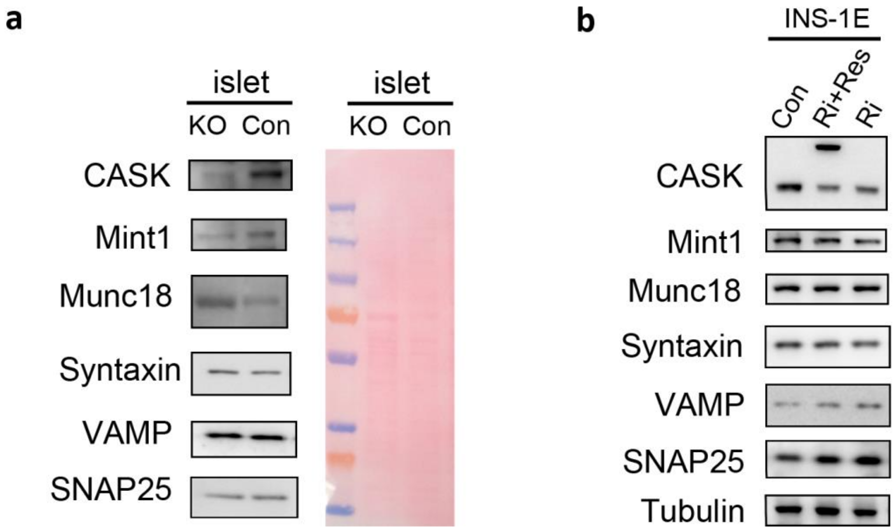

Supplementary Fig. S3 The expression levels of secretory machinery proteins in CASK-depleted islets and INS-1E cells Western blot showing the expression levels of some important proteins of secretory machinery in CASK knockout (KO) and control islets (a) and differently treated INS-1E cells (b). Con, cells were transfected with non-targeting siRNA plus empty vector; Ri, cells were treated with siRNA targeting CASK; Ri+Res, CASK knockdown cells were transfected with siRNA-resistant CASK.

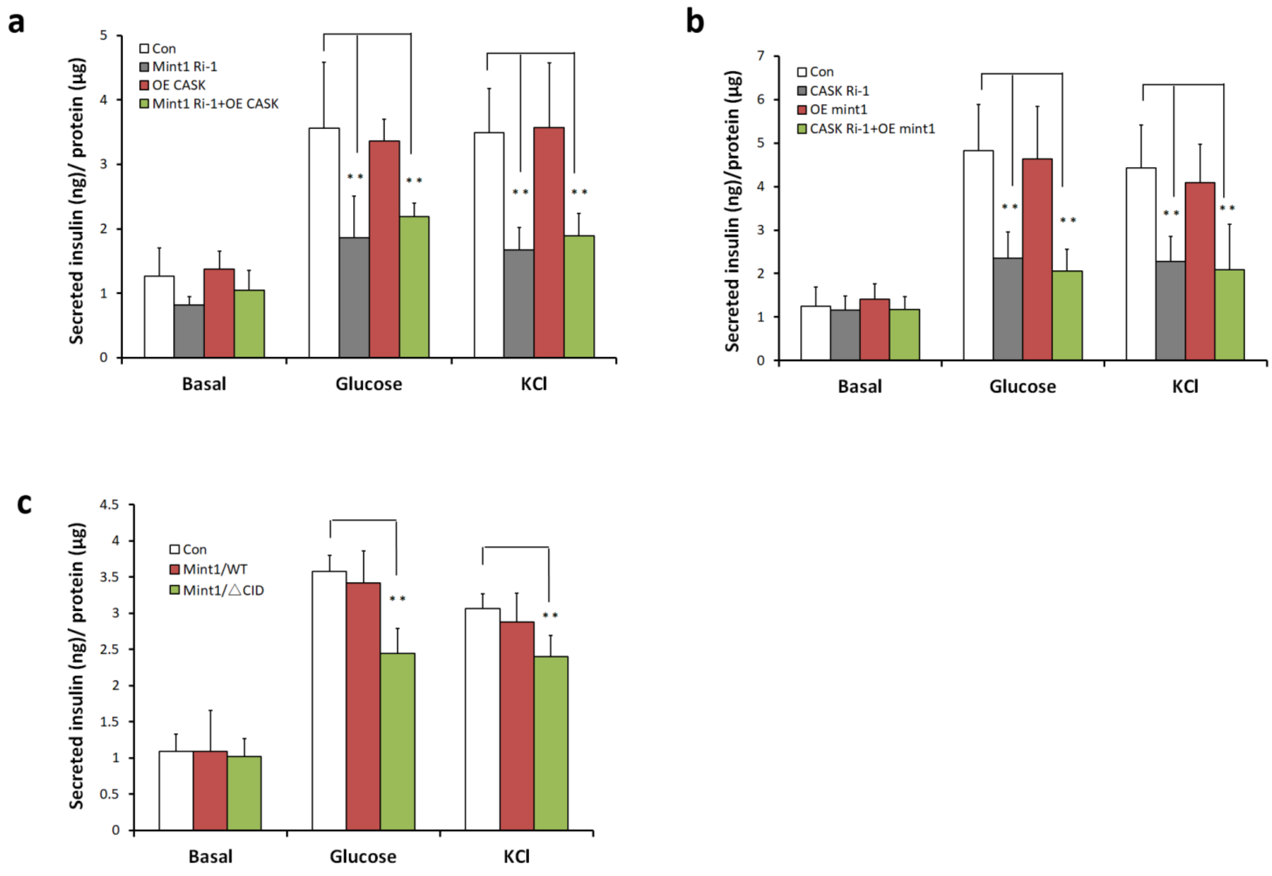

Supplementary Fig. S4 CASK co-operate with Mint1 to regulate insulin secretion in INS-1E cells (a) Reduced insulin secretion due to RNAi targeting Mint1 (Mint1 Ri-1) could not be rescued by overexpressing CASK (OE CASK). (b) Reduced insulin secretion due to RNAi targeting CASK (CASK Ri-1) could not be rescued by over-expressing Mint (OE Mint1). (c) The effects of over-expressing Mint1 mutant lacking CASK-interacting domain (Mint1/△CID) on insulin secretion.

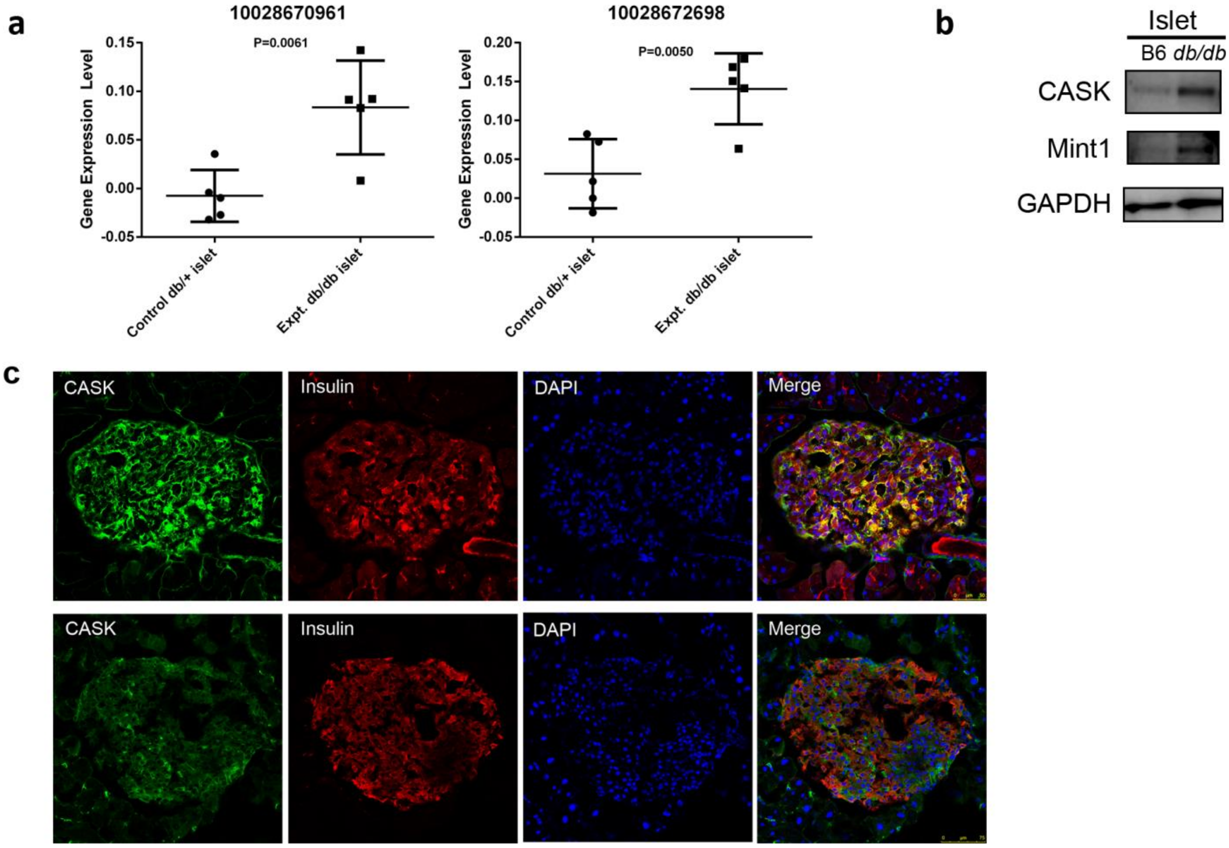

Supplementary Fig. S5 CASK/Mint1 expression is upregulated in early stage of diabetes (a) The plot of CASK gene expression levels in Control db/+ and db/db islet samples. The bottom and top bands indicate the standard deviation (SD) of the expression levels; the band in the middle is the mean. The expression levels of CASK were measured by two probe sets with accession numbers: 10028670961 and 10028672698. n=5. (b) Western blotting showing the expression of CASK and Mint1 in control (B6) and db/db islet. (c) Immunofluorescence staining demonstrating the expression of CASK in B6 and db/db islet. For each assay, three mice were analyzed.

.

Supplementary Table S1 Data collection and structural refinement statistics

**Data collection**	
Data set	CASK(1-319)/Mint1(338-397)
Beam Line	BL17U1
Wavelength (Å)	0.979
Space group	$P2_12_12_1$
Cell dimensions	
a, *b*, *c* (Å)	93.98, 96.32, 97.56
α, β, γ (°)	90.00, 90.00, 90.00
Resolution range (Å)	50.00-2.35 (2.43-2.35) [a]
No. Reflections	37889 (3725)
R_{merge} (%) [b]	12.10 (51.80)
$I / \sigma I$	12.60 (5.80)
Completeness (%)	99.9 (100)
Redundancy	10.1(10.2)
**Strucutre Refinement**	
Resolution (Å)	42.23 – 2.40 (2.47 – 2.40)
No. Reflections R_{work}/R_{free}	35239/1737
R_{work} (%) [c] / R_{free} (%) [d]	18.82 (20.43) / 22.41 (25.87)
No. atoms	
Protein	5875
Water	152
B-factors	47.0
R.m.s. deviations	
Bond lengths (Å)	0.009
Bond angles (°)	1.231
Ramachandran plot (%) (favored/additional/disallowed)	98.61/1.39/0.00
Maximum likelihood coordinate error	0.21

a. The values in parentheses refer to the highest resolution shell.

b. R_{merge} = 100 ΣhΣi|Ih, I —〈Ih〉|/ΣhΣiIh, i, where the outer sum (h) is over the unique reflections and the inner sum (i) is over the set of independent observations of each unique reflection.

c. R_{work} is the R_{factor} for the working dataset. $R_{factor}=\Sigma||Fo|-|Fc||/\Sigma|Fo|$ where $|Fo|$ and $|Fc|$ are observed and calculated structure factor amplitudes respectively.

d. R_{free} is the cross-validation R_{factor} computed for a randomly chosen subset of 4.93% of the total number of reflections, which were not used during refinement.

Supplemental Methods

Expression constructs

Full length human CASK in pRK5/myc (kindly provided by Dr. B. Margolis, University of Michigan Medical School) was subcloned into pEGFP-C1 vector (Clontech). Full length of rat Mint1 was amplified from rat brain cDNA using Takara PrimeSTAR® HS (Premix) and cloned into pCDNA3-JFLAG vector (kindly provided by Dr. A. Ullrich, Germany). Mint1/4A mutant (W381A, I355A, I359A, V362A) and Mint1 siRNA-resistant mutants were constructed using the KOD -Plus- Mutagenesis Kit (TOYOBO). Mint1/ΔCID mutant (331-432a.a.deleted) and CASK/ΔCAMK mutant (8-307a.a.deleted) were constructed from the full length constructs. Rat Munc18-1a (kindly provided by Dr. David James, Garvan Institute of Medical Research) was subcloned into pCDNA3-HA vector (Clontech). All constructs were verified by DNA sequencing. Primers are listed in the following table.

Plasmid name	Primers
Mint1/WT	Forward:5'-CGGGGTACCGATGAACCACTTGGAGGGCTC-3'<br>Reverse:5'-GCTCTAGAGATGTAAACGGGCTGCTCCTGG-3'
Mint1/W381A	Forward:5'-GCGGTCATGCGCCAGGACATTAGCC-3'<br>Reverse:5'-GATGGGCTCTTTGGGTTCGTCGGGG-3'
Mint1/I355A,I359A,V362A	Forward:5'-GCCGAAGAAGCGAAAACCAGGACC-3'<br>Reverse:5'-GGCCTCCTTGGCGTCCTTGATGGCC-3'
Mint1/ΔCID	Forward:5'-GGTTCCCACAACTCGCTGCTGCTGC-3'<br>Reverse:5'-AAAGAGTCAAGAAAAAGCTTGGCTTC-3'
Mint1 siRNA-resistant	Forward:5'-CGATCTTATCCACTTCTCCAAGTCG-3'<br>Forward:5'-TCGTTGTACATGTCCTGGGTGTTG-3'
CASK/ΔCAMK	Forward:5'-GTACTAGCCGCTGTGTCAAGTCACA-3'<br>Reverse:5'-CAGCACGTCGTCGTCGGCCAT-3'
Munc18-1a	Forward:5'-GGAATTCTG ATGGCCCCCATTGGCCTCAAGGCG-3'<br>Reverse:5'-GCTCTAGA TTAACTGCTTATTTCTTCGTCTG-3'
CASK siRNA-resistant	Forward: 5'-GATCCAGCCAAGGACGATCTCATACCCTGTAAAGAAGCTGG-3'<br>Reverse:5'-CCAGCTTCTTTACAGGGTATGAGATCGTCCTTGGCTGGATC-3'

Transfection

Lipofectamine LTX (Invitrogen) or electroporation was used to introduce exogenous DNA into INS-1E cells. The DNA Transfection Reagent (Biotool) was used to introduce exogenous DNA into 293 cells. All the transfections were performed according to the manufacturer's instructions.

Silencing of genes using small interfering RNA

The following siRNAs were used in this study: two siRNAs targeting rat CASK, 5- GGATGACCTCATCCCCTGC-3 and 5-GGATCGTTATGCCTACAAA-3, named CASK Ri-1 and CASK Ri-2 respectively; two siRNAs targeting Mint1, 5-GTACAACGATGACCTGATC-3, and 5-CTTGATCGATGGAATTATT-3, named Mint-1 Ri-1 and Mint-1 Ri-2 respectively; non-silencing control siRNA used as a negative control, 5 -TTCTCCGAACGTGTCACGT-3. The siRNAs were transiently transfected into INS-1E cells using lipofectamine RNAiMAX (Invitrogen) according to the manufacturer's instructions. For silencing CASK in rat islets, the islets were infected with adenoviruses expressing shRNA harboring the same siRNA sequence as CASK Ri-1 or shRNA containing the same non-silencing control siRNA sequence.

Immunoprecipitation and Western Blotting

Cells were harvested in HTNG buffer (20 mM HEPES, pH 7.4, 150 mM NaCl, 10% glycerol, 1%Triton X). Total protein amount was quantified using the BCA Protein Assay Kit (Tiangen). Cell lysates were incubated with the indicated antibodies and protein-A/G Sepharose (GE Healthcare) overnight at 4°C. Beads were washed and eluted with SDS loading buffer. Samples were then subjected to SDS-PAGE (4-12% Bis-Tris gel), transferred to a nitrocellulose membrane, and analyzed by immunoblotting with the indicated antibodies. The following antibodies were used in these assays: monoclonal anti-HA (M20003, 1:100,00), monoclonal anti-GFP (M20004, 1:5,000) and anti-Flag (M20008L, 1:5,000) were purchased from Abmart; polyclonal anti-GFP (ab290, 1:5,000) was from Abcam; monoclonal anti-CASK (610782), anti-Munc18-1 (610337) and anti-Ecadherin (610181) were from BD Biosciences; monoclonal anti-tubulin (T5168, 1:5,000), anti-syntaxin-1 (S0664, 1:5,000) was from Sigma-Aldrich; polyclonal anti-VAMP2 (104202) was from Synaptic Systems; monoclonal anti-GAPDH (KC-5G4, 1:5,000) was from Kangcheng, polyclonal anti-LaminB1 (sc-20682) was from Santa Cruz; polyclonal anti-CASK, polyclonal anti-Mint1, and monoclonal anti-Mint1 were prepared in our own laboratory. All the antibodies were used at 1:1000 dilution unless mentioned otherwise.

Islet size
Bright-field images of isolated islets were obtained using Leica TCS SP2 microscope (Leica, Germany). Islet area was analyzed using ImageJ software.

Insulin secretion assay
INS-1E cells were incubated with RPMI 1640 medium, lacking glucose (Thermo) for 2hr at 37°C. After 20min subsequent incubation in Krebs-Ringer bicarbonate HEPES buffer (KRBH) (pH 7.4) containing 10 mM HEPES, 0.1% BSA, 128 mM NaCl, 4.8 mM KCl, 2.5 mM CaCl2, 1.2 mM MgSO4, 1.2 mM KH2PO4, and 5 mM NaHCO3, cells were stimulated with KRBH containing 2.5mM glucose, 20mM glucose or 30mM KCl for 1hr at 37°C. Conditioned media were then collected. After a serial centrifugation at 500 g for 10min, 14000 g for 15 min, insulin levels in the supernatant were measured by ELISA assay (Rat Insulin ELISA Kit, Alpco). Intracellular insulin was extracted by adding 75% ethanol containing 0.18 N HCl overnight at 4°C, followed by ELISA assay (Rat Insulin ELISA Kit, Alpco).

CASK-floxed mice$^{(flx+/+)}$ were crossed with RIP-Cre transgene mice$^{(RIP\text{-}Cre+)}$ (Cre) to generate β-cell-specific *CASK* knockout mice$^{(flx+/+;RIP\text{-}Cre+)}$. Islets were isolated from the pancreas of CASK-knockout and control mice (RIP-Cre transgene mice) (8-12 weeks) as described before (1). Hanks' balanced salt solution containing collagenase P (Roche) was injected into the pancreas via the common bile duct. The pancreas then was excised and was digested by incubation at 37 C for 15 min. Islets were semipurified by two centrifugation steps at 290 g for 1min at 4 °C, followed by centrifugation on a Ficoll discontinuous gradient (Sigma-Aldrich). Islets were manually harvested from this semipure preparation. Before insulin release experiments, islets were allowed to recover for 24 h in RPMI 1640 medium containing 10% fetal calf serum, 11 mM glucose, 100 IU penicillin, and 0.1 mg/ml streptomycin. To measure insulin release from islets in static incubations, islets of comparable size were preincubated for 30 min in KRBH buffer. Released insulin was collected after incubating the islets for 60 min in KRBH with 2.5 mM glucose, 25 mM glucose or 2.5 mM glucose plus 30 mM KCl. Supernatants were collected and centrifuged as described for cells. Insulin levels in the supernatant were measured by ELISA (Ultra Sensitive Mouse Insulin ELISA Kit, Crystal Chem) following the manufacturer's instruction.

To analyze insulin release kinetics, a customized perfusion apparatus was used (BioRep). Islets of similar size in KRBH buffer were loaded into columns with Bio-Gel P-4Gell (Bio-Rad) and pre-incubated with 2.5 mM glucose in KRBH buffer for 45 min, with a constant flow of 100 μl/min. After the prerun, the islets were exposed sequentially to 2.5 mM glucose (10 min), 25 mM glucose (25 min), 2.5 mM glucose plus 30 mM KCl (5 min). The flow-through was collected in a 96 well plate (1 min per well). To measure total insulin content of the isolated islets, islets were sonicated to obtain islet homogenate, and then incubated with 75% ethanol containing 0.18 N HCl overnight at 4°C. Protein content in islet homogenate was measured using BCA Protein Assay Kit (Tiangen) according to the manufacture's instruction. Insulin levels were measured using Ultra Sensitive Mouse Insulin ELISA Kit (Crystal Chem) according to the manufacturer's instruction.

Immunofluorescence microscopy
Cells were fixed with cold methanol. Samples of mouse pancreas tissue were fixed with 4% paraformaldehdye, and cryoprotected with 30% sucrose in PBS. Tissues were frozen at -80°C and cut into 5-μm sections on a sliding microtome (Leica). Cover slips were mounted using Fluorescence Mounting Medium (DAKO). Nuclei were counterstained with DAPI. The following antibodies were used in immunofluorescent assay: monoclonal anti-CASK (prepared in-house, 1:200), polyclonal anti-CASK (SC-10777, 1:200, Sata cruz), monoclonal anti-Munc18-1 (610337, 1:100, BD Biosciences), polyclonal anti-insulin (I2018, 1:1,000, Sigma-Aldrich), monoclonal anti-glucagon (G2654, 1:1,000, Sigma-Aldrich), monoclonal anti-somatostatin (MAB354, 1:100, Chemicon), Alexa Fluor 568 goat anti-rabbit, Alexa Fluor 568 goat anti-mouse, Alexa Fluor 488 goat anti-rabbit, and Alexa Fluor 488 goat anti-mouse IgG (all at 1:1,000, Invitrogen). Images were photographed using a fluorescent confocal microscope (Zeiss LSM710, Germany).

Analytical gel-filtration chromatography
Analytical gel-filtration chromatography was carried out on an ÄKTA FPLC system (GE Healthcare). Purified CASK-CaMK, Mint1-MID-CID, Munc18-1 or the mixture of three proteins was concentrated to ~2.0 mg/ml (OD_{280}) separately and 100μl sample was applied to an analytical gel-filtration Superdex-200 10/300 GL column (GE Healthcare) equilibrated with buffer (50 mM Tris, pH 8.0, 100 mM NaCl, 1 mM EDTA, and 1 mM DTT). The flow rate applied was 0.5 ml/min, and 0.5 ml fractions were collected. The column was standardized using Bio-Rad's gel-filtration standard mixture of Ferritin (Mr 440 kDa, with an elution volume of 11.3 ml), BSA (Mr 134 and 67 kDa, with elution volume of 12.8 and 14.9 ml respectively), β-lactoglobulin (Mr 35 kDa, with an elution volume of 16.1 ml), and Cytochrome C (Mr 13.6 kDa, with a elution volume of 18 ml). The elution volumes of molecular weight markers were indicated at the top.

Isothermal titration calorimetry (ITC) assay
ITC was carried out on a MicroCalorimeter ITC200 (Microcal LLC) at 25°C. All proteins were dissolved in a buffer

containing 50 mM Tris(pH 8.0), 100 mM NaCl,1 mM EDTA and 1 mM DTT. The titration processes were performed by injecting 20× 2 μl aliquots of protein sample in a syringe (concentration of ~250-350 μM) into the stirred protein sample in the calorimeter cell (concentration of ~25-40 μM) at time intervals of 120 s to ensure that the titration peak returned to baseline. Each experiment was repeated three times. The heat of dilution obtained by the titration of the syringe sample into the buffer was subtracted. The data were analyzed using ORIGIN 8.0 and fitted by the one-site-binding model.

Structural determination

The initial phase was determined by molecular replacement using the structure models of CASK-CaMK (PDB code: 3TAC) as the searching models with PHASER (2).An incomplete structure model was further manually built with COOT (3) and refined with PHENIX (4) against the 2.45 Å data set. The Mint1-CID was built manually according to the 2Fo-Fc and Fo-Fc electron density maps. In the final stage, an additional TLS refinement was performed in PHENIX. The overall quality of the final structural models of the CASK/Mint1 complex was assessed by PROCHECK(5). Sequence alignments were prepared using CLUSTAL-W (6) .The statistics for the data collection and structural refinement were summarized in Table S1. Atomic coordinates and structure factors have been deposited in the Protein Data Bank.

1. Atasoy D*, et al.* (2007) Deletion of CASK in mice is lethal and impairs synaptic function. *Proc Natl Acad Sci U S A* 104(7):2525-2530.
2. McCoy AJ (2007) Solving structures of protein complexes by molecular replacement with Phaser. *Acta crystallographica. Section D, Biological crystallography* 63(Pt 1):32-41.
3. Emsley P & Cowtan K (2004) Coot: model-building tools for molecular graphics. *Acta crystallographica. Section D, Biological crystallography* 60(Pt 12 Pt 1):2126-2132.
4. Adams PD*, et al.* (2010) PHENIX: a comprehensive Python-based system for macromolecular structure solution. *Acta crystallographica. Section D, Biological crystallography* 66(Pt 2):213-221.
5. Laskowski RA, Macarthur MW, Moss DS, & Thornton JM (1993) Procheck - a Program to Check the Stereochemical Quality of Protein Structures. *J Appl Crystallogr* 26:283-291.
6. Thompson JD, Higgins DG, & Gibson TJ (1994) CLUSTAL W: improving the sensitivity of progressive multiple sequence alignment through sequence weighting, position-specific gap penalties and weight matrix choice. *Nucleic acids research* 22(22):4673-4680.
